# Cochrane's risk of bias tool for non-randomized studies (ROBINS-I) is frequently misapplied: A methodological systematic review

**DOI:** 10.1016/j.jclinepi.2021.08.022

**Published:** 2021-12

**Authors:** Erik Igelström, Mhairi Campbell, Peter Craig, Srinivasa Vittal Katikireddi

**Affiliations:** MRC/CSO Social and Public Health Sciences Unit, University of Glasgow, Berkeley Square 99 Berkeley Street**, Glasgow,** G3 7HR

**Keywords:** Risk of bias, Systematic review methods, Cochrane, Non-randomized studies, Observational studies

## Abstract

**Objectives:**

We aimed to review how ‘Risk of Bias In Non-randomized Studies–of Interventions’ (ROBINS-I), a Cochrane risk of bias assessment tool, has been used in recent systematic reviews.

**Study Design and Setting:**

Database and citation searches were conducted in March 2020 to identify recently published reviews using ROBINS-I. Reported ROBINS-I assessments and data on how ROBINS-I was used were extracted from each review. Methodological quality of reviews was assessed using AMSTAR 2 (‘A MeaSurement Tool to Assess systematic Reviews’).

**Results:**

Of 181 hits, 124 reviews were included. Risk of bias was serious/critical in 54% of assessments on average, most commonly due to confounding. Quality of reviews was mostly low, and modifications and incorrect use of ROBINS-I were common, with 20% reviews modifying the rating scale, 20% understating overall risk of bias, and 19% including critical-risk of bias studies in evidence synthesis. Poorly conducted reviews were more likely to report low/moderate risk of bias (predicted probability 57% [95% CI: 47–67] in critically low-quality reviews, 31% [19–46] in high/moderate-quality reviews).

**Conclusion:**

Low-quality reviews frequently apply ROBINS-I incorrectly, and may thus inappropriately include or give too much weight to uncertain evidence. Readers should be aware that such problems can lead to incorrect conclusions in reviews.

**What is new?**

**Key findings**
• In a sample of systematic reviews published across two months in 2020 (*n* = 124) that used the ROBINS-I tool to assess risk of bias, authors often modified or the tool or used it incorrectly. Poorly conducted reviews were more likely to rate risk of bias as low and moderate and less likely to rate it as critical, compared to well-conducted reviews.
• Risk of bias was rated as serious or critical in most studies, with the ‘confounding’ bias domain most often rated highly.

**What this adds to what was known?**
• This is the first study to investigate how ROBINS-I has been used in practice in the systematic review literature.
• Cochrane recommend that ROBINS-I should only be used by review teams with extensive methodological expertise, but our findings suggest that the tool is nonetheless frequently misapplied.

**What is the implication and what should change now?**
• Inadequate risk of bias assessment can substantively affect the findings of a systematic review, potentially leading to misleading recommendations and guidelines. Researchers and practitioners should be alert to this issue when reading systematic reviews, and authors should ensure that the conduct and reporting of risk of bias assessments are rigorous.

## 1 Introduction

Non-randomized studies of interventions (NRSIs) are an essential source of evidence in many fields where the effectiveness of interventions is of interest, but randomized controlled trials (RCTs) are not always feasible for practical or ethical reasons. NRSIs can achieve high precision when large data sets are used, but are susceptible to more complex and powerful biases than RCTs [1,2]. This presents challenges when evidence from NRSIs is included in systematic reviews and clinical guidelines.

‘Risk of Bias In Non-randomized Studies – of Interventions’ (ROBINS-I) is a tool for assessing the risk of bias (RoB) in NRSIs, which was published in 2016 after 5 years of development and piloting [3,4]. Although ROBINS-I was designed to assess clinical intervention studies, modifications for other types of non-randomized study (NRS) are under development [5,6]. Unlike earlier appraisal tools, which tended to focus on identifying methodological flaws in specific study designs [7,8], ROBINS-I integrates an understanding of causal inference based on counterfactual reasoning [9], [10], [11]. Using this theoretical foundation, ROBINS-I assesses RoB on an absolute scale for the causal inferences of any NRSI regardless of study design, where ‘low’ RoB is comparable to a well-conducted RCT. ROBINS-I has been seen as a significant methodological innovation and a step change in how NRSIs can be utilized in evidence syntheses [12], [13], [14].

ROBINS-I is the only tool recommended by the Cochrane Handbook for assessing RoB in NRSIs[15], and has been increasingly widely adopted in published and planned systematic reviews[16]. However, there are concerns that the tool's conceptual complexity may have made it challenging to use, and that many review teams may lack the necessary expertise to apply it correctly [12], [17], [18]. In areas of public health where uncertain evidence from NRSIs is common, there is also a concern that ROBINS-I may be of limited use due to the lack of discrimination among higher levels of RoB [12], [19].

Previous methodological studies examining ROBINS-I have relied on small research teams applying the tool in a controlled setting [12], [17], [18], [20], [21], [22]. However, no research has yet been undertaken on how ROBINS-I has been used in the large and growing body of reviews using the tool in practice. The aim of this study was to review how ROBINS-I has been used in recent systematic reviews, in order to identify ways that it may be misapplied and possible explanatory factors.

## 2 Methods

### 2.1 Search strategy

A protocol for this study was developed prospectively and registered in PROSPERO (CRD42013006924; submitted 8 March, registered 11 June 2020). Searches were carried out in Scopus, Web of Science, MEDLINE, Embase, and the Cochrane Database of Systematic Reviews (CDSR). Title and abstract searches were conducted in all databases using the following search terms:

“risk of bias in non randomi#ed studies of interventions” OR “risk of bias in nonrandomi#ed studies of interventions” OR “robins i”

Where possible (in all databases except Embase and CDSR), forward citation searches were also conducted to identify publications that cited the ROBINS-I tool[4].

The searches were carried out on 2 March 2020, and were limited to publications from 1 January 2020 onwards. This time window of approximately 2 months was selected to balance a sufficient number of reviews against available time and resources, and to provide an understanding of contemporary research practice.

### 2.2 Selection of included reviews

Publications were included if they were presented by the authors as systematic reviews, reported that ROBINS-I was used to assess RoB, and were available in English. Search results were independently screened by two reviewers (E.I. and S.V.K.) and any disagreements resolved by consensus.

### 2.3 Data extraction

The outcome of interest was the ROBINS-I RoB assessments reported within each review. Each outcome in an included study is assigned judgments in seven ‘bias domains’, as well as an overall RoB judgment[4]. The possible judgments are ‘low’, ‘moderate’, ‘serious’, ‘critical’, and ‘no information’.

Reported ROBINS-I assessments within each review were extracted using a Microsoft Excel spreadsheet. Where separate assessments were reported for multiple results or outcomes within a single study, assessments for each result were extracted separately. In addition to these assessments, the following information was extracted for each review: aim or research question as described by the authors, number of included studies (RCTs and NRSs), whether meta-analysis was performed, and any declared funding sources or competing interests. Additional information was also captured on how ROBINS-I was used and reported, including whether assessments were performed in duplicate, whether results from the pre-assessment stage were reported, in what form ROBINS-I assessments for individual studies were reported, and what categories were used for the assessments. Free-form textual comments were captured regarding non-standard uses of ROBINS-I, modifications to the tool, and any other noteworthy observations, including quotations from reviews where relevant.

Data extraction was carried out by E.I. and review-level data were checked by a second reviewer (S.V.K., P.C., or M.C.).

### 2.4 Quality appraisal

The methodological quality of the included reviews was assessed using AMSTAR 2 (‘A MeaSurement Tool to Assess systematic Reviews 2’)[2]. This tool consists of 16 items, each of which is rated as ‘yes’, ‘partial yes’, or ‘no’. Each item is regarded as either ‘critical’ or ‘non-critical’, and an overall confidence rating can be determined by the number of identified weaknesses (i.e., ‘no’ ratings) in critical and non-critical domains. Overall confidence in a review is rated ‘critically low’ if it has more than one critical flaw, ‘low’ if it has exactly one critical flaw, ‘moderate’ if it has no critical flaws but more than one non-critical weakness, and ‘high’ if it has no critical flaws and up to one non-critical weakness.

Quality appraisals were conducted independently by two reviewers (E.I., M.C., P.C., and S.V.K.), and disagreements were resolved by consensus.

### 2.5 Analysis

Free-text comments on non-standard use of ROBINS-I were grouped into categories and summarized. For each review, we calculated the proportion of judgments in each RoB category and the range of categories used.

For reviews that did not report overall RoB judgments, inferred overall judgments were calculated by taking the highest judgment in an individual domain, in accordance with the ROBINS-I guidance.[4] For reviews that reported both domain-specific and overall judgments, the inferred overall judgment was compared to the reported judgment to identify instances where they differed.

We considered the following explanatory variables as potential predictors of RoB judgments: methodological quality as assessed using AMSTAR 2, whether RoB assessment was performed in duplicate, whether the authors reported industry funding or competing interests, and whether the review included RCTs.

Since the RoB assessments within the same review were expected to be similar, multilevel regression was used. The overall (or inferred overall) RoB judgments were treated as an ordinal outcome, and a separate generalized ordered logit model was fitted for each predictor with a review-level random intercept. Results were presented as odds ratios and population-average marginal predicted probabilities. Analyses were carried out in Stata MP/16.1[23] using the *gllamm* command[24].

## 3 Results

**Fig. 1:**
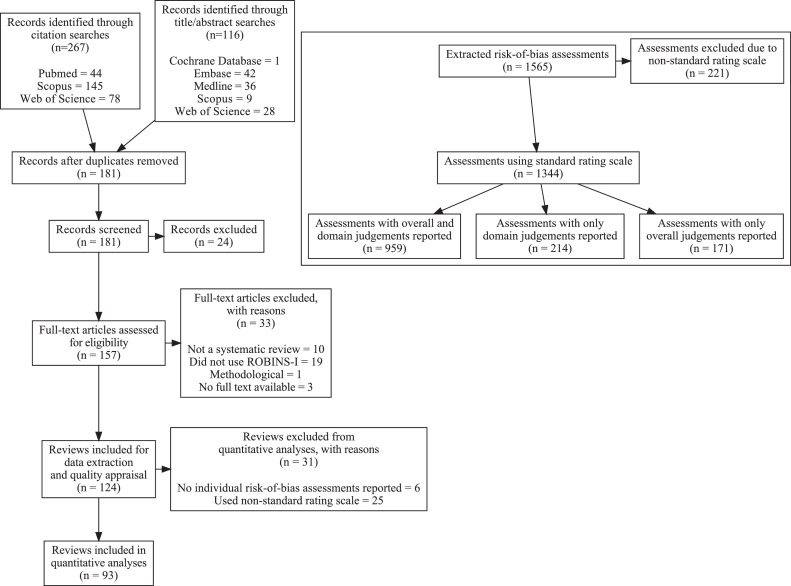
Adapted Preferred Reporting Items in Systematic Reviews and Meta-Analyses (PRISMA) flow diagram showing the study selection process. Top right: Flow diagram illustrating the risk of bias assessments included in the quantitative analyses.

The literature search identified 124 systematic reviews satisfying the inclusion criteria (Fig. 1). The results covered a wide range of topics (Table 1), with most reviews (66%) studying individual-level clinical interventions. Methodological quality was rated as low or critically low in most reviews, with only 17% and 6% being classified as moderate and high quality, respectively. A complete list of included reviews and excluded full-text articles can be found in Supplementary Tables 1 and 2.

**Table 1:** Characteristics of the included systematic reviews

		AMSTAR 2 confidence rating			
Characteristic	Overall, N=124	High, N = 7 (6%)	Moderate, N = 21 (17%)	Low, N = 41 (33%)	Critically low, N = 55 (44%)
Type of intervention studied					
Clinical intervention (drug)	26 (21%)	0 (0%)	5 (24%)	12 (29%)	9 (16%)
Clinical intervention (other)	30 (24%)	0 (0%)	9 (43%)	8 (20%)	13 (24%)
Clinical intervention (surgical)	26 (21%)	1 (14%)	1 (5%)	7 (17%)	17 (31%)
Environmental exposure	4 (3%)	2 (29%)	0 (0%)	1 (2%)	1 (2%)
Non-clinical intervention	3 (2%)	0 (0%)	2 (10%)	1 (2%)	0 (0%)
Public health intervention	21 (17%)	2 (29%)	1 (5%)	11 (27%)	7 (13%)
Non-interventional	14 (11%)	2 (29%)	3 (14%)	1 (2%)	8 (15%)
Number of included studies					
Median (IQR)	14 (9, 27)	14 (8, 22)	16 (7, 36)	15 (10, 27)	13 (9, 24)
Range	2, 124	3, 29	2, 91	4, 47	2, 124
Funding sources					
Academic	2 (2%)	0 (0%)	0 (0%)	0 (0%)	2 (4%)
Foundation/NGO	9 (7%)	0 (0%)	1 (5%)	4 (10%)	4 (7%)
Government	23 (19%)	2 (29%)	4 (19%)	8 (20%)	9 (16%)
Industry	4 (3%)	1 (14%)	0 (0%)	1 (2%)	2 (4%)
Multiple (excl. industry)	11 (9%)	2 (29%)	3 (14%)	3 (7%)	3 (5%)
Multiple (incl. industry)	2 (2%)	0 (0%)	0 (0%)	2 (5%)	0 (0%)
Other	2 (2%)	0 (0%)	0 (0%)	2 (5%)	0 (0%)
None	44 (35%)	2 (29%)	9 (43%)	15 (37%)	18 (33%)
Not reported	27 (22%)	0 (0%)	4 (19%)	6 (15%)	17 (31%)
Competing interests					
Academic	11 (9%)	2 (29%)	2 (10%)	5 (12%)	2 (4%)
Industry	15 (12%)	1 (14%)	1 (5%)	7 (17%)	6 (11%)
None	92 (74%)	4 (57%)	16 (76%)	26 (63%)	46 (84%)
Not reported	6 (5%)	0 (0%)	2 (10%)	3 (7%)	1 (2%)
Review included randomized trials	70 (56%)	2 (29%)	16 (76%)	21 (51%)	31 (56%)
Review included meta-analysis	73 (59%)	5 (71%)	7 (33%)	20 (49%)	41 (75%)

**Table 2:** How risk of bias assessments using ROBINS-I were conducted and reported in the included systematic reviews

		AMSTAR 2 confidence rating			
Characteristic	Overall, N = 124	High, N = 7	Moderate, N = 21	Low, N = 41	Critically low, N = 55
ROBINS-I judgments reported					
Overall and domain-specific RoB judgments for all studies	82 (66%)	7 (100%)	15 (71%)	27 (66%)	33 (60%)
Domain-specific RoB judgments for all studies	19 (15%)	0 (0%)	3 (14%)	5 (12%)	11 (20%)
Overall RoB judgments for all studies	15 (12%)	0 (0%)	2 (10%)	7 (17%)	6 (11%)
Incomplete or aggregated RoB judgments only	2 (2%)	0 (0%)	0 (0%)	0 (0%)	2 (4%)
No RoB judgments	6 (5%)	0 (0%)	1 (5%)	2 (5%)	3 (5%)
Reported justifications for individual ROBINS-I judgments					
Overall and domain-specific RoB judgments for all studies	6 (5%)	2 (29%)	1 (5%)	3 (7%)	0 (0%)
Domain-specific RoB judgments for all studies	5 (4%)	0 (0%)	0 (0%)	2 (5%)	3 (5%)
Overall RoB judgments for all studies	3 (2%)	0 (0%)	0 (0%)	3 (7%)	0 (0%)
No justifications reported	110 (89%)	5 (71%)	20 (95%)	33 (80%)	52 (95%)
Reporting of ROBINS-I pre-assessment stage					
Confounding domains listed	5 (4%)	0 (0%)	4 (19%)	1 (2%)	0 (0%)
Confounding domains and co-interventions listed	3 (2%)	1 (14%)	1 (5%)	1 (2%)	0 (0%)
No reporting of pre-assessment stage	116 (94%)	6 (86%)	16 (76%)	39 (95%)	55 (100%)
Scale used to rate RoB					
Standard scale (low, moderate, serious, critical)	93 (75%)	5 (71%)	16 (76%)	31 (76%)	41 (75%)
Non-standard: 3 levels (e.g., low, moderate, high)	14 (11%)	1 (14%)	2 (10%)	6 (15%)	5 (9%)
Non-standard: 2 levels (e.g., low, high)	9 (7%)	1 (14%)	2 (10%)	2 (5%)	4 (7%)
Non-standard: other (e.g., yes, probably yes, probably no, no)	2 (2%)	0 (0%)	0 (0%)	0 (0%)	2 (4%)
None	6 (5%)	0 (0%)	1 (5%)	2 (5%)	3 (5%)
Explicitly incorporated RoB into evidence synthesis					
Yes	38 (31%)	6 (86%)	13 (62%)	13 (32%)	6 (11%)
No	86 (69%)	1 (14%)	8 (38%)	28 (68%)	49 (89%)
How RoB was incorporated into evidence synthesis (N = 38)					
Discussed RoB in narrative synthesis	28 (74%)	5 (83%)	11 (85%)	9 (69%)	3 (50%)
Conducted sensitivity analysis or subgroup analysis	10 (26%)	3 (50%)	3 (23%)	3 (23%)	1 (17%)
Excluded studies at high RoB	7 (18%)	1 (17%)	2 (15%)	2 (15%)	2 (33%)
Deviations from ROBINS-I guidance					
Modified the rating scale	25 (20%)	2 (29%)	4 (19%)	8 (20%)	11 (20%)
Modified the bias domains	8 (6%)	0 (0%)	1 (5%)	2 (5%)	5 (9%)
Assigned an overall RoB judgment lower than the highest-rated bias domain^a^	18 (20%)	0 (0%)	5 (31%)	7 (23%)	6 (17%)
Included critical-RoB studies any synthesis	24 (19%)	2 (29%)	7 (33%)	10 (24%)	5 (9%)
Included critical-RoB studies in meta-analysis	9 (7%)	1 (14%)	2 (10%)	2 (5%)	4 (7%)
Applied ROBINS-I to a non-interventional research question	14 (11%)	2 (29%)	3 (14%)	1 (2%)	8 (15%)

RoB, risk of bias.

a Excluding studies that did not report both overall and per-domain RoB judgements. N=90

### 3.1 Reporting of risk of bias judgments

Although most reviews (66%) reported both the overall RoB judgments and judgments for each individual domain, a considerable number omitted either or both. Most reviews (89%) reported only the judgments without any supporting comments or justifications: only six reviews (5%) reported explicit justifications for both overall and per-domain RoB judgments. The pre-assessment stage of ROBINS-I, where relevant confounding domains and co-interventions are identified, was not described at all in 94% of reviews; only eight reviews (6%) explicitly listed what confounding domains were considered.

### 3.2 Modifications and non-standard use of ROBINS-I

Modifications and non-standard uses of the tool were common, often without explanation or justification. These are described in Table 2, and in greater detail in Supplementary Table 3. Modifications of the rating scale were most common, occurring in 20% of reviews. Only one of these justified the use of a non-standard scale: Rhodes et al. [25] used ‘low risk’, ‘some concerns’, and ‘high risk’, stating that ‘[these] terms were used to be concordant with the randomized trials’ (which were assessed using the Cochrane RoB 2 tool [26]). In the remaining reviews, no justification was provided.

Most reviews used the seven bias domains listed in the tool. In five reviews, one domain was omitted or reported as ‘not applicable’ (domain 3, ‘classification of interventions’ [27] or 4, ‘deviations from intended interventions’ [28], [29], [30], [31]). One review used all seven domains, but added an eighth domain (‘vested interest bias’) [32]. One review appeared to use the seven ROBINS-I domains, but reported the results alongside results from the Cochrane RoB tool for RCTs, with domains renamed to fit both tools [33].

Most reviews (69%) did not make references to RoB when synthesizing evidence (Table 2). Among the reviews that did, most referred to RoB in individual studies in narrative discussion or interpretation of results, and a smaller number incorporated RoB into meta-analysis (e.g., using subgroup analysis or sensitivity analysis) or excluded studies at high RoB. Contrary to guidance [4], [34]. In 19% of reviews, studies rated at critical RoB were included in the narrative or quantitative synthesis; 7% included critical-RoB studies in a meta-analysis. Also contrary to guidance, 11% of reviews applied ROBINS-I to a non-interventional research question (e.g., estimating incidence or prevalence of a condition [35], [36] or describing the features of a population [37], [38], [39], [40], [41]).

ROBINS-I guidance states that the overall RoB judgment should normally be at least as high as the highest judgment in any bias domain[4]. Out of the 90 reviews that reported both overall and domain-specific judgments, 20% did not adhere to this principle, and thus not all studies or results reported as low RoB overall were rated low in all domains. In the 68 reviews that reported judgments for all domains using the standard scale, 58% of the studies or results reported as low RoB overall had higher RoB in at least one domain, and 27% (all from a single review [31]) had critical RoB in at least one domain (Supplementary Table 4).

### 3.3 Distribution of risk of bias judgments

Individual ROBINS-I assessments were extracted from 116 reviews, comprising a total of 1,565 assessments of 1,499 primary studies (Fig. 1). 221 assessments from 23 reviews used a non-standard rating scale and were excluded from further analysis. Of the assessments that remained, 171 only included an overall judgment, and 214 only included domain-specific judgments. For the latter, an inferred overall judgment was calculated by taking the highest RoB judgment in an individual domain. The final analyzed sample included 1,344 RoB assessments from 93 reviews.

**Fig. 2:**
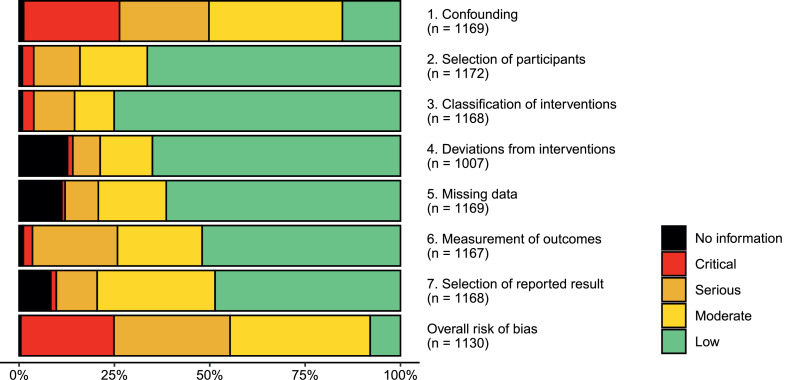
Distribution of risk of bias judgments in each bias domain for ROBINS-I assessments within the included systematic reviews. For interpretation of the references to color in this figure legend, the reader is referred to the Web version of this article.

Among the 93 reviews reporting judgments using the standard scale, RoB was reported as low in 10% of studies on average, moderate in 36%, and critical in 15% (Table 3). However, these proportions varied by methodological quality as assessed using AMSTAR 2, with more well-conducted reviews containing fewer low- and moderate-RoB studies and more critical-RoB studies on average. Out of the reviews that included five or more NRSs, most made use of two (45%) or three (40%) adjacent categories; 13% of reviews assigned the same overall judgment to all included NRSs. Out of the seven bias domains, domain 1 (confounding) was more often rated at higher RoB than the remaining domains (Fig. 2), and was among the highest-rated domains in 74% of all the included NRSs. The pattern was similar across different AMSTAR 2 confidence ratings (Supplementary Figure 2).

**Table 3:** Reported ROBINS-I risk of bias judgments in included systematic reviews that used the standard ROBINS-I scale

		AMSTAR 2 confidence rating			
Characteristic	Overall, N = 93	High, N = 5	Moderate, N = 16	Low, N = 31	Critically low, N = 41
Mean (SD) proportion of studies rated					
Low	0.10 (0.23)	0.02 (0.03)	0.10 (0.26)	0.08 (0.24)	0.12 (0.23)
Moderate	0.36 (0.33)	0.22 (0.40)	0.27 (0.25)	0.34 (0.32)	0.41 (0.35)
Serious	0.39 (0.33)	0.44 (0.44)	0.28 (0.25)	0.45 (0.31)	0.38 (0.35)
Critical	0.15 (0.29)	0.32 (0.41)	0.34 (0.42)	0.11 (0.23)	0.08 (0.23)
No information	0.01 (0.03)	0.00 (0.00)	0.00 (0.02)	0.01 (0.02)	0.01 (0.04)
Range of risk of bias categories used (reviews with >5 studies only)					
One category (all studies rated the same)	9 (13%)	0 (0%)	0 (0%)	3 (12%)	6 (21%)
Two categories	30 (45%)	2 (50%)	4 (36%)	11 (46%)	13 (46%)
Three categories	27 (40%)	2 (50%)	7 (64%)	10 (42%)	8 (29%)
Four categories	1 (1%)	0 (0%)	0 (0%)	0 (0%)	1 (4%)
(5 or fewer studies)	26	1	5	7	13
Type of intervention studied					
Clinical intervention (drug)	23 (25%)	0 (0%)	5 (31%)	11 (35%)	7 (17%)
Clinical intervention (other)	25 (27%)	0 (0%)	7 (44%)	7 (23%)	11 (27%)
Clinical intervention (surgical)	17 (18%)	0 (0%)	0 (0%)	6 (19%)	11 (27%)
Environmental exposure	3 (3%)	2 (40%)	0 (0%)	0 (0%)	1 (2%)
Non-clinical intervention	3 (3%)	0 (0%)	2 (12%)	1 (3%)	0 (0%)
Non-interventional	11 (12%)	1 (20%)	2 (12%)	1 (3%)	7 (17%)
Public health intervention	11 (12%)	2 (40%)	0 (0%)	5 (16%)	4 (10%)
Number of included studies					
Median (IQR)	14 (9, 29)	14 (9, 27)	16 (11, 40)	15 (10, 27)	12 (8, 28)
Range	2, 124	3, 29	3, 91	4, 40	2, 124
Review included randomized trials	53 (57%)	1 (20%)	12 (75%)	17 (55%)	23 (56%)
Review included meta-analysis	58 (62%)	4 (80%)	6 (38%)	16 (52%)	32 (78%)

Excludes reviews not reporting risk of bias using the standard ROBINS-I categories (low, moderate, serious, critical, no information)

### 3.4 Associations between review characteristics and risk of bias judgments

As Table 3 shows, reviews of higher methodological quality (as assessed using AMSTAR 2) appeared to have a lower proportion of low/moderate overall RoB judgments and a higher proportion of critical judgments, with an apparent dose-response relationship. The multilevel regression analyses confirmed this (Table 4), with the predicted probability of low RoB judgments ranging from 5% (95% CI: 2%–11%) in high/moderate-quality reviews to 13% (8%–20%) in critically low-quality reviews. Probability of moderate and lower RoB similarly ranged from 31% (19%–46%) in high/moderate-quality reviews to 57% (47%–67%) in critically low-quality reviews, and critical RoB was approximately twice as likely in moderate/high-quality reviews (30% [17%–45%]) as in low-quality (13% [7%–22%]) or critically low-quality reviews (15% [9%–23%]).

**Table 4:** Associations between review characteristics and overall ROBINS-I risk of bias judgements reported in the included systematic reviews

	Odds ratio (95% CI)			Marginal predicted probability, % (95% CI)			N studies (reviews)
	Low vs. higher RoB	Low/moderate vs. higher RoB	Critical vs. lower RoB	Low RoB	Low/moderate RoB	Critical RoB	
Review quality^a^							
High/Moderate (baseline)				5 (2, 11)	31 (19, 46)	30 (17, 45)	426 (21)
Low	1.9 (0.36, 10.0)	3.3 (0.78, 14)	0.19 (0.04, 0.82)	8 (4, 14)	46 (35, 58)	13 (7, 22)	409 (31)
Critically low	4.7 (1.0, 22)	7.7 (1.9, 31)	0.24 (0.06, 0.98)	13 (8, 20)	57 (47, 67)	15 (9, 23)	504 (41)
Review included RCTs^a^							
No (baseline)				12 (7, 18)	45 (34, 56)	19 (13, 28)	701 (40)
Yes	0.59 (0.17, 2.0)	1.4 (0.47, 4.4)	0.69 (0.21, 2.2)	9 (5, 14)	50 (40, 60)	16 (10, 23)	638 (53)
RoB assessed in duplicate^a^							
Yes (baseline)				11 (7, 16)	47 (38, 55)	22 (15, 29)	992 (63)
No	0.34 (0.08, 1.5)	1.3 (0.40, 4.4)	0.20 (0.05, 0.70)	6 (2, 12)	51 (38, 63)	10 (5, 16)	347 (30)
Industry funding or competing interests^b^							
No (baseline)				14 (9, 20)	50 (42, 58)	17 (12, 23)	795 (62)
Yes	<0.01 (<0.01, 0.07)	0.48 (0.10, 2.3)	0.56 (0.10, 3.1)	0 (0, 3)	41 (23, 60)	13 (5, 29)	231 (13)

CI, confidence interval; RoB, risk of bias.

A separate generalized ordered logit regression model was fitted for each predictor, with a review-level random intercept.

a N = 1339 studies, 93 reviews.

b N = 1026 studies, 75 reviews.

There was less conclusive evidence of any association between RoB judgments and duplicate ROBINS-I assessment, reported industry funding/competing interests, or the inclusion of RCTs in the review. Although there were strong associations between duplicate RoB assessment and the proportion of critical RoB judgments, as well as between industry funding and low RoB judgments, the direction and strength of association were not consistent across levels of RoB.

## 4 Discussion

In a representative sample of 124 systematic reviews that used ROBINS-I to assess RoB in NRSs, methodological quality (assessed using AMSTAR 2) was generally low, unjustified modifications of ROBINS-I were common, and reporting of RoB assessments were often lacking in detail. Reported RoB was generally high, with confounding being by far the most common reason for high RoB judgemnts. However, low-quality reviews were more likely to report low or moderate RoB (where low RoB is interpreted as comparable to a well-conducted RCT [4]), and less likely to report critical RoB.

### 4.1 Strengths and limitations

This is the first study investigating the practical use of ROBINS-I in a sizeable sample of published reviews from a range of disciplines. Previous methodological studies of ROBINS-I have been conducted on a relatively small scale, with assessments conducted by the authors themselves, and including a limited number of studies [12], [17], [18], [21], [22]. By using multilevel methods on a comparatively large sample of individual ROBINS-I assessments, we have achieved greater statistical power than would have been possible with review-level data alone. However, it is possible that the associations seen in the quantitative analyses may be confounded by review characteristics, such as topic area or discipline.

In line with previous literature employing AMSTAR 2 to study review quality [42], [43], [44], [45], only a small proportion of included reviews were of high methodological quality, and only one was a Cochrane review. Hence, this study provides limited insight into how ROBINS-I has been employed in the most well-conducted reviews. We were also unable to include any reviews not available in English, although since this was checked at the screening stage, and no reviews were excluded for this reason, this did not affect the results. Due to resource limitations, we were also unable to conduct independent data extraction, although review selection and quality assessment were conducted in duplicate, and extracted data were checked by a second reviewer.

The lack of detailed reporting on RoB assessments in most reviews was also a limiting factor. Since only a small number of reviews included explicit justifications for their ROBINS-I judgments, it was not possible to examine how far these were plausible or justified, as has been done in similar methodological reviews studying the Cochrane RoB tool for RCTs [46], [47]. Assessments from a substantial number of reviews also had to be excluded from quantitative analyses because they used a rating scale that deviated too far from the official tool.

### 4.2 Interpretation of results

The distribution of RoB judgments we observed is largely in line with previous studies. Losilla et al. [21], Thomson et al. [12], and Dhiman et al. [22] reported overall RoB of at least moderate, serious, and serious respectively, used no more than two adjacent RoB categories, and reported that the confounding domain usually determined the overall judgment. Minozzi et al. [17] reported statistics for inter-rater reliability, but no individual RoB judgments. Our results confirm that similar patterns can be seen in published reviews.

Unexplained modifications of the tool and departures from guidance were common. This is not unexpected, given previous evidence that other RoB tools are often inadequately applied [46], [47]. It is mostly unclear whether such departures were deliberate or in error, but some may clearly have impacted the results of a review in ways that could mislead a reader. For example, a majority of studies reported as having low RoB would have been given a more conservative judgment if the recommended process for determining an overall RoB judgment had been followed. Additionally, although ROBINS-I was explicitly designed to assess RoB in NRSIs, several reviews inappropriately applied the tool to NRSs with non-interventional research questions. This may reflect an unmet need for equally rigorous appraisal tools that can be applied to non-interventional study designs[6].

We found that poorly conducted reviews were more likely to rate included studies as having low RoB. Assuming that poorly conducted reviews were more likely to have applied ROBINS-I incorrectly, this can be taken as evidence that incorrect application of ROBINS-I led authors to understate the true RoB on average. Although the association between review quality and RoB judgments could be confounded by review characteristics, we found no indication that low-quality reviews were systematically different from high-quality reviews with respect to topic or other features that might otherwise explain the differences in reported RoB. The reasons for any potential underestimation of RoB are unclear, given the lack of detailed reporting in the included reviews, but given that substantial expertise is needed to apply ROBINS-I correctly, it is plausible that less experienced or thorough review teams are likely to overlook subtle or complex sources of bias that a team with greater epidemiological expertise would identify.

### 4.3 Practical implications

As has been emphasized in previous literature [4], [15], [34] ROBINS-I is a complex tool, and should only be used by review teams with extensive methodological expertise and sufficient time to commit to learning how to apply the tool correctly. Our findings suggest that ROBINS-I is nonetheless often applied with insufficient rigour, and consequently RoB may be underestimated in many reviews that include NRSs.

Our findings highlight that substantial expertise in epidemiology and systematic review methods is required when conducting reviews that include NRSs. The Cochrane Handbook emphasizes the importance of assessing RoB in seven key domains, with ROBINS-I being the only tool presented that explicitly does this [15], [34] and this may have encouraged some review authors to use ROBINS-I despite lacking the necessary expertise. Authors should carefully consider the trade-off between rigour and ease-of-use in different tools: although more easily-applied tools are available (e.g., the Newcastle-Ottawa scale), these often fail to cover important bias domains, and hence potentially fail to identify sources of bias that a more rigorous tool could have identified [34], [48]. At the same time, a more rigorous tool could give a false impression of certainty if it is not rigorously applied. Many alternative appraisal tools also fail to distinguish clearly between risk of bias and external validity [7].

Since RoB ratings directly influence which studies are included in a meta-analysis or narrative synthesis, the accuracy of RoB assessments can have a direct impact on the results of a review. We thus recommend that readers carefully consider whether RoB assessments were conducted rigorously and correctly. Current appraisal tools [2], [49] and reporting guidelines [50] for systematic reviews make few explicit recommendations about how much detail is appropriate when reporting the results of RoB assessments. When using ROBINS-I, we recommend that authors report the output of the pre-assessment stage (including important confounding domains and co-interventions), both overall and domain-specific RoB judgments, and written justifications for at least the overall judgment.

## 5 Conclusion

ROBINS-I appears to be frequently misused by systematic reviews of low methodological quality, which often modify the tool or deviate from its official guidance. Low-quality reviews are more likely to underestimate RoB, and may thus inappropriately include or give too much weight to uncertain evidence. Readers should be aware that such problems can lead to incorrect conclusions in reviews. Authors should identify the RoB assessment tool most suitable for their review, and be aware that correct application of the ROBINS-I tool requires expertise and sufficient resources.

## CRediT author statement

Erik Igelström: conceptualization, methodology, investigation, formal analysis, writing – original draft. Mhairi Campbell: validation (quality appraisal), writing – review & editing. Peter Craig: validation (quality appraisal), writing – review & editing. S. Vittal Katikireddi: conceptualization, supervision, validation (quality appraisal), writing – review & editing.

## Appendix Supplementary materials

Image, application 1
