# Supplementary material for: Cochrane's risk of bias tool for non-randomized studies (ROBINS-I) is frequently misapplied: A methodological systematic review
Source: J Clin Epidemiol. 2021 Dec;140:22–32. doi: 10.1016/j.jclinepi.2021.08.022 (PMC8809341; doi:10.1016/j.jclinepi.2021.08.022)

Cochrane's risk-of-bias tool for non-randomised studies (ROBINS-I) is frequently misapplied: a methodological systematic review

Supplementary Tables and Figures

Erik Igelström, Mhairi Campbell, Peter Craig, S. Vittal Katikireddi

Contents

Supplementary Table 1: List of included systematic reviews . . . 2

Supplementary Table 2: Publications excluded after full-text screening . . . 10

Supplementary Table 3: Characteristics of the included systematic reviews . . . 13

Supplementary Table 4: Modifications and non-standard uses of ROBINS-I . . . 17

Supplementary Table 5: Overall ROBINS-I risk-of-bias judgements compared to the highest judgement in an individual domain . . . 23

Supplementary Figure 1: Summary of quality ratings of the included systematic reviews for each individual item in the AMSTAR 2 tool . . . 24

Supplementary Figure 2: Distribution of risk-of-bias judgements in each bias domain for studies assessed using ROBINS-I within the included systematic reviews, stratified by the AMSTAR 2 confidence rating of the review . . . 25

Supplementary Table 1: List of included systematic reviews

	Reference	DOI
1	Agnelli G, Belch JJF, Baumgartner I, Giovas P, Hoffmann U. Morbidity and mortality associated with atherosclerotic peripheral artery disease: A systematic review. Atherosclerosis. 2020 Jan;293:94–100.	doi:10.1016/j.atherosclerosis.2019.09.012
2	Alshafei A, Cascio S, Boland F, O'Shea N, Hickey A, Quinn F. Comparing the outcomes of tubularized incised plate urethroplasty and dorsal inlay graft urethroplasty in children with hypospadias: A systematic review and meta-analysis. J Pediatr Urol. 2020 Apr;16(2):154–61.	doi:10.1016/j.jpurol.2020.01.009
3	Arn M-L, Dritsas K, Pandis N, Kloukos D. The effects of fixed orthodontic retainers on periodontal health: A systematic review. Am J Orthod Dentofacial Orthop. 2020 Feb;157(2):156–164.e17.	doi:10.1016/j.ajodo.2019.10.010
4	Baker BS, Stannard MS, Duren DL, Cook JL, Stannard JP. Does blood flow restriction therapy in patients older than age 50 result in muscle hypertrophy, increased strength, or greater physical function? A systematic review. Clin Orthop. 2020 Dec;478(3):593–606.	doi:10.1097/CORR.0000000000001090
5	Balla A, Alarcon I, Morales-Conde S. Minimally invasive component separation technique for large ventral hernia: Which is the best choice? A systematic literature review. Surg Endosc. 2020 Oct;34(1):14–30.	doi:10.1007/s00464-019-07156-4
6	Bang CS, Yang YJ, Lee JJ, Baik GH. Endoscopic submucosal dissection of early gastric cancer with mixed-type histology: A systematic review. Dig Dis Sci. 2020 Jul;65(1):276–91.	doi:10.1007/s10620-019-05761-w
7	Belachew A, Tewabe T, Dessie G. Neonatal mortality and its association with antenatal care visits among live births in Ethiopia: A systematic review and meta-analysis. J Matern Fetal Neonatal Med. 2020 Jan;1–8.	doi:10.1080/14767058.2020.1718093
8	Belk JW, Kraeutler MJ, Houck DA, Smith JR, McCarty EC. Comparing hamstring autograft with hybrid graft for anterior cruciate ligament reconstruction: A systematic review. Arthroscopy. 2020 Apr;36(4):1189–201.	doi:10.1016/j.arthro.2019.10.010
9	Bellos I, Kontzoglou K, Perrea DN. Predictors of tolvaptan short-term response in patients with refractory ascites: A meta-analysis. J Gastroenterol Hepatol. 2020 Aug;35(2):182–91.	doi:10.1111/jgh.14784
10	Bezeczky Z, El-Banna A, Petrou S, Kemp A, Scourfield J, Forrester D, et al. Intensive family preservation services to prevent out-of-home placement of children: A systematic review and meta-analysis. Child Abuse Negl. 2020 Apr;102:104394.	doi:10.1016/j.chiabu.2020.104394
11	Brennan SE, McDonald S, Page MJ, Reid J, Ward S, Forbes AB, et al. Long-term effects of alcohol consumption on cognitive function: A systematic review and dose-response analysis of evidence published between 2007 and 2018. Syst Rev. 2020 Feb;9(1):33.	doi:10.1186/s13643-019-1220-4
12	Brouwer RJ, Lalieu RC, Hoencamp R, Hulst RA van, Ubbink DT. A systematic review and meta-analysis of hyperbaric oxygen therapy for diabetic foot ulcers with arterial insufficiency. J Vasc Surg. 2020 Feb;71(2):682–692.e1.	doi:10.1016/j.jvs.2019.07.082
13	Cardoso PC, Espinosa DG, Mecenas P, Flores-Mir C, Normando D. Pain level between clear aligners and fixed appliances: A systematic review. Prog Orthod. 2020 Jan;21(1):3.	doi:10.1186/s40510-019-0303-z
14	Carrier FM, Chass M, Wang HT, Aslanian P, Iorio S, Bilodeau M, et al. Restrictive fluid management strategies and outcomes in liver transplantation: A systematic review. Can J Anaesth. 2020 Sep;67(1):109–27.	doi:10.1007/s12630-019-01480-y
15	Carter P, Fou L, Whiter F, Delgado Nunes V, Hasler E, Austin C, et al. Management of mesh complications following surgery for stress urinary incontinence or pelvic organ prolapse: A systematic review. BJOG. 2020 Nov;127(1):28–35.	doi:10.1111/1471-0528.15958
16	Checcucci E, De Cillis S, Porpiglia F. 3D-printed models and virtual reality as new tools for image-guided robot-assisted nephron-sparing surgery: A systematic review of the newest evidences. Curr Opin Urol. 2020 Jan;30(1):55–64.	doi:10.1097/MOU.0000000000000686

	Reference	DOI
17	Checcucci E, Veccia A, Fiori C, Amparore D, Manfredi M, Di Dio M, et al. Retzius-sparing robot-assisted radical prostatectomy vs the standard approach: A systematic review and analysis of comparative outcomes. BJU Int. 2020 Aug;125(1):8–16.	doi:10.1111/bju.14887
18	Chen J-Y, Wei S-K, Lai C-C, Weng T-S, Wang H-H. A metaanalysis comparing the efficacy and safety of peramivir with other neuraminidase inhibitors for influenza treatment. Medicina (Kaunas). 2020 Feb;56(2):63.	doi:10.3390/medicina56020063
19	Coates D, Makris A, Catling C, Henry A, Scarf V, Watts N, et al. A systematic scoping review of clinical indications for induction of labour. Mastrolia SA, editor. PLoS One. 2020 Jan;15(1):e0228196.	doi:10.1371/journal.pone.0228196
20	Cutts JC, Agius PA, Lin Z, Powell R, Moore K, Draper B, et al. Pregnancy-specific malarial immunity and risk of malaria in pregnancy and adverse birth outcomes: A systematic review. BMC Med. 2020 Jan;18(1).	doi:10.1186/s12916-019-1467-6
21	Daugaard CL, Hangaard S, Bartels EM, Gudbergsen H, Christensen R, Bliddal H, et al. The effects of weight loss on imaging outcomes in osteoarthritis of the hip or knee in people who are overweight or obese: A systematic review. Osteoarthritis Cartilage. 2020;28:10–21.	doi:10.1016/j.joca.2019.10.013
22	Davidson B, Schoen N, Sedighim S, Haldenby R, Dalziel B, Breitbart S, et al. Intrathecal baclofen versus selective dorsal rhizotomy for children with cerebral palsy who are nonambulant: A systematic review. J Neurosurg Pediatr. 2020;25(1):69–77.	doi:10.3171/2019.8.PEDS19282
23	DeNicola N, Grossman D, Marko K, Sonalkar S, Butler Tobah YS, Ganju N, et al. Telehealth interventions to improve obstetric and gynecologic health outcomes: A systematic review. Obstet Gynecol. 2020;135(2):371–82.	doi:10.1097/AOG.0000000000003646
24	Di Giuseppe G, Thacker N, Schechter T, Pole JD. Anxiety, depression, and mental health-related quality of life in survivors of pediatric allogeneic hematopoietic stem cell transplantation: A systematic review. Bone Marrow Transplant. 2020 Jan;55(7):1240–54.	doi:10.1038/s41409-020-0782-z
25	Djade CD, Porgo TV, Zomahoun HTV, Perrault-Sullivan G, Dionne CE. Incidence of shoulder pain in 40 years old and over and associated factors: A systematic review. Eur J Pain. 2020 Oct;24(1):39–50.	doi:10.1002/ejp.1482
26	Djarv T, Swain JM, Chang WT, Zideman DA, Singletary E. Early or first aid administration versus late or in-hospital administration of aspirin for non-traumatic adult chest pain: A systematic review. Cureus. 2020 Feb;12.	doi:10.7759/cureus.6862
27	Falk Brekke A, Overgaard S, Hrobjartsson A, Holsgaard-Larsen A. Non-surgical interventions for excessive anterior pelvic tilt in symptomatic and non-symptomatic adults: A systematic review. EFORT Open Rev. 2020 Jan;5(1):37–45.	doi:10.1302/2058-5241.5.190017
28	Fang EH, Wang XL, Feng JX, Zhao X. The prognostic role of Glasgow prognostic score and C-reactive protein to albumin ratio for sarcoma: A system review and meta-analysis. Dis Markers. 2020 Jan;2020:1–4.	doi:10.1155/2020/8736509
29	Ford ME, Groet E, Daams JG, Geurtsen GJ, Van Bennekom CAM, Van Someren EJW. Non-pharmacological treatment for insomnia following acquired brain injury: A systematic review. Sleep Med Rev. 2020;50:101255.	doi:10.1016/j.smrv.2019.101255
30	Frascella B, Oradini-Alacreu A, Balzarini F, Signorelli C, Lopalco PL, Odone A. Effectiveness of email-based reminders to increase vaccine uptake: A systematic review. Vaccine. 2020 Jan;38(3):433–43.	doi:10.1016/j.vaccine.2019.10.089
31	Froggatt S, Covey J, Reissland N. Infant neurobehavioural consequences of prenatal cigarette exposure: A systematic review and meta-analysis. Acta Paediatr. 2020 Jan;109(6):1112–24.	doi:10.1111/apa.15132
32	Gagelmann N, Ayuk F, Atanackovic D, Kroger N. B cell maturation antigen-specific chimeric antigen receptor T cells for relapsed or refractory multiple myeloma: A meta-analysis. Eur J Haematol. 2020 Jan;104(4):318–27.	doi:10.1111/ejh.13380
33	Gandolla M, Antonietti A, Longatelli V, Pedrocchi A. The effectiveness of wearable upper limb assistive devices in degenerative neuromuscular diseases: A systematic review and meta-analysis. Front Bioeng Biotechnol. 2019;7:450.	doi:10.3389/fbioe.2019.00450
34	Garbazza C, Hackethal S, Riccardi S, Cajochen C, Cicolin A, D'Agostino A, et al. Polysomnographic features of pregnancy: A systematic review. Sleep Med Rev. 2020 Apr;50:101249.	doi:10.1016/j.smrv.2019.101249

	Reference	DOI
35	Geierlehner A, Rodi T, Mosahebi A, Tanos G, Wormald JCR. Meta-analysis of venous anastomosis techniques in free flap reconstruction. J Plast Reconstr Aesthet Surg. 2020 Mar;73(3):409–20.	doi:10.1016/j.bjps.2019.11.033
36	Gentile D, Donadon M, Lleo A, Aghemo A, Roncalli M, Tommaso L di, et al. Surgical treatment of hepatocholangiocarcinoma: A systematic review. Liver Cancer. 2020 Nov;9(1):15–27.	doi:10.1159/000503719
37	Giannopoulos S, Texakalidis P, Charisis N, Jonnalagadda AK, Chaitidis N, Giannopoulos S, et al. Synchronous carotid endarterectomy and coronary artery bypass graft versus staged carotid artery stenting and coronary artery bypass graft for patients with concomitant severe coronary and carotid stenosis: A systematic review and meta-analysis. Ann Vasc Surg. 2020 Jan;62:463–473.e4.	doi:10.1016/j.avsg.2019.06.018
38	Giles AE, Valencia M, Parpia S, Fu E, Ruo L, Simunovic M, et al. Simultaneous versus staged resection of rectal cancer and synchronous liver metastases (RESECT): A systematic review and meta-analysis. Eur Surg. 2020 Apr;52(1):15–8.	doi:10.1007/s10353-019-0582-0
39	Gogou M, Pavlou E, Haidopoulou K. Therapies that are available and under development for Duchenne muscular dystrophy: What about lung function? Pediatr Pulmonol. 2020;55:300–15.	doi:10.1002/ppul.24605
40	Goldshtein D, Krensky C, Doshi S, Perelman VS. In situ simulation and its effects on patient outcomes: A systematic review. BMJ Simul Technol Enhanc Learn. 2020;6:3–9.	doi:10.1136/bmjstel-2018-000387
41	Gottlieb M, Dyer S, Peksa GD. Beta-blockade for the treatment of cardiac arrest due to ventricular fibrillation or pulseless ventricular tachycardia: A systematic review and meta-analysis. Resuscitation. 2020 Jan;146:118–25.	doi:10.1016/j.resuscitation.2019.11.019
42	Graham EA, Deschnes SS, Khalil MN, Danna S, Filion KB, Schmitz N. Measures of depression and risk of type 2 diabetes: A systematic review and meta-analysis. J Affect Disord. 2020 Mar;265:224–32.	doi:10.1016/j.jad.2020.01.053
43	Guo L, Feng S, Sun B, Jiang X, Liu Y. Benefit and risk profile of tofacitinib for the treatment of alopecia areata: A systemic review and meta-analysis. J Eur Acad Dermatol Venereol. 2020;34:192–201.	doi:10.1111/jdv.15937
44	Gupta D, Potter TD, Disher T, Eaton K, Goldstein L, Patel L, et al. Comparative effectiveness of catheter ablation devices in the treatment of atrial fibrillation: A network meta-analysis. J Comp Eff Res. 2020;9:115–26.	doi:10.2217/cer-2019-0165
45	Hajibandeh S, Maw A. Diathermy versus scalpel for skin incision in patients undergoing open inguinal hernia repair: A systematic review and meta-analysis. Int J Surg. 2020 Mar;75:35–43.	doi:10.1016/j.ijsu.2020.01.020
46	Hajibandeh S, Dave M, Tarazi M, Satyadas T. Laparoscopic versus open liver resection for tumors in the posterosuperior segments: A systematic review and meta-analysis. Surg Laparosc Endosc Percutan Tech. 2020;30(2):93–105.	doi:10.1097/SLE.0000000000000746
47	Handel MN, Rohde JF, Jacobsen R, Nielsen SM, Christensen R, Alexander DD, et al. Processed meat intake and incidence of colorectal cancer: A systematic review and meta-analysis of prospective observational studies. Eur J Clin Nutr. 2020 Feb;74:1132–48.	doi:10.1038/s41430-020-0576-9
48	Hasperhoven GF, Al-Nasiry S, Bekker V, Villamor E, Kramer BWW. Universal screening versus risk-based protocols for antibiotic prophylaxis during childbirth to prevent early-onset Group B streptococcal disease: A systematic review and meta-analysis. BJOG. 2020 Feb;127(6):680–91.	doi:10.1111/1471-0528.16085
49	Heesen M, Hilber N, Rijs K, Marel C van der, Rossaint R, Schffer L, et al. Intrathecal catheterisation after observed accidental dural puncture in labouring women: Update of a meta-analysis and a trial-sequential analysis. Int J Obstet Anesth. 2020;41:71–82.	doi:10.1016/j.ijoa.2019.08.001
50	Hsu C-Y, Chen C-Y, Lin Y-M, Tam K-W. Efficacy and safety of high-dose vs low-dose leucovorin in patients with colorectal cancer: Systematic review and meta-analysis. Colorectal Dis. 2020 Jul;22(1):17–6.	doi:10.1111/codi.14746

	Reference	DOI
51	Hu RQ, Velthoven MH van, Meinert E. Perspectives of people who are overweight and obese on using wearable technology for weight management: Systematic review. JMIR Mhealth Uhealth. 2020 Jan;8(1):e12651.	doi:10.2196/12651
52	Huang YS, Zheng Q, Yang H, Fu X, Zhang X, Xia C, et al. Efficacy of intermittent or continuous very low-energy diets in overweight and obese individuals with type 2 diabetes mellitus: A systematic review and meta-analyses. J Diabetes Res. 2020 Jan;2020:1–21.	doi:10.1155/2020/4851671
53	Indarwati F, Mathew S, Munday J, Keogh S. Incidence of peripheral intravenous catheter failure and complications in paediatric patients: Systematic review and meta analysis. Int J Nurs Stud. 2020;102:103488.	doi:10.1016/j.ijnurstu.2019.103488
54	Jassal M, Egan G, Dahri K. Opioid prescribing in the elderly: A systematic review. J Pharm Technol. 2020 Aug;36(1):28–40.	doi:10.1177/8755122519867975
55	Kamfose MM, Muriithi FG, Knight T, Lasserson D, Hayward G. Intravenous ceftriaxone versus multiple dosing regimes of intravenous anti-staphylococcal antibiotics for methicillin-susceptible Staphylococcus aureus (MSSA): A systematic review. Antibiotics (Basel). 2020 Jan;9(2):39.	doi:10.3390/antibiotics9020039
56	Karamini A, Bakopoulou A, Andreadis D, Gkiouras K, Kritis A. Therapeutic potential of mesenchymal stromal stem cells in rheumatoid arthritis: A systematic review of in vivo studies. Stem Cell Rev Rep. 2020 Jan;16(2):276–87.	doi:10.1007/s12015-020-09954-z
57	Kling K, Harder T, Younger Z, Burchard G, Schmidt-Chanasit J, Wichmann O. Vaccination against Japanese encephalitis with IC51: Systematic review on immunogenicity, duration of protection, and safety. J Travel Med. 2020 Feb;27(2):taaa016.	doi:10.1093/jtm/taaa016
58	Koenig C, Schneider C, Morgan JE, Ammann RA, Sung L, Phillips B. Association of time to antibiotics and clinical outcomes in patients with fever and neutropenia during chemotherapy for cancer: A systematic review. Support Care Cancer. 2020 Jul;28(3):1369–83.	doi:10.1007/s00520-019-04961-4
59	Kokkinidis DG, Arfaras-Melainis A, Giannopoulos S, Katsaros I, Jawaid O, Jonnalagadda AK, et al. Statin therapy for reduction of cardiovascular and limb-related events in critical limb ischemia: A systematic review and meta-analysis. Vasc Med. 2020 Jan;25(2):106–17.	doi:10.1177/1358863X19894055
60	Kolding L, Eken H, Uldbjerg N. Drug exposure during pregnancy and fetal cardiac function - a systematic review. J Perinat Med. 2020 Mar;48(3):199–208.	doi:10.1515/jpm-2019-0402
61	Kowalewski M, Raffa GM, Zielinski K, Alanazi M, Gilbers M, Heuts S, et al. The impact of Centre's heart transplant status and volume on in-hospital outcomes following extracorporeal membrane oxygenation for refractory post-cardiotomy cardiogenic shock: A meta-analysis. BMC Cardiovasc Disord. 2020 Jan;20(1):10.	doi:10.1186/s12872-019-01317-y
62	Lam PPY, Du RN, Peng SM, McGrath CPJ, Yiu CKY. Oral health status of children and adolescents with autism spectrum disorder: A systematic review of case-control studies and meta-analysis. Autism. 2020 Jan;24(5):136236131987733.	doi:10.1177/1362361319877337
63	Leach MJ, Jones M, Bressington D, Jones A, Nolan F, Muyambi K, et al. The association between community mental health nursing and hospital admissions for people with serious mental illness: A systematic review. Syst Rev. 2020;9(1):35.	doi:10.1186/s13643-020-01292-y
64	Lee SI, Patel M, Dutton B, Weng S, Luveta J, Qureshi N. Effectiveness of interventions to identify and manage patients with familial cancer risk in primary care: A systematic review. J Community Genet. 2020 May;11(1):73–83.	doi:10.1007/s12687-019-00419-6
65	Lee H, Ryu K, Sohn Y, Kim J, Suh GY, Kim E. Impact on patient outcomes of pharmacist participation in multidisciplinary critical care teams: A systematic review and meta-analysis. Crit Care Med. 2020 Sep;47(9):1243–50.	doi:10.1097/CCM.0000000000003830
66	Lee JA, Levy DA, Patel KG, Brennan E, Oyer SL. Hair transplantation in frontal fibrosing alopecia and lichen planopilaris: A systematic review. Laryngoscope. 2020 Feb;131(1):59–66.	doi:10.1002/lary.28551
67	Liu S, Gnjidic D, Nguyen J, Penm J. Effectiveness of interventions on the appropriate use of opioids for noncancer pain among hospital inpatients: A systematic review. Br J Clin Pharmacol. 2020 Jan;86(2):210–43.	doi:10.1111/bcp.14203

	Reference	DOI
68	Louwers A, Warnink-Kavelaars J, Daams J, Beelen A. Effects of upper extremity surgery on activities and participation of children with cerebral palsy: A systematic review. Dev Med Child Neurol. 2020 Jul;62(1):21–7.	doi:10.1111/dmcn.14315
69	Lu J, Lu Y, Xun Y, Chen F, Wang S, Cao S. Impact of endourological procedures with or without double-J stent on sexual function: A systematic review and meta-analysis. BMC Urol. 2020;20(1):13.	doi:10.1186/s12894-020-0582-1
70	Lussier ME, Evans HJ, Wright EA, Gionfriddo MR. The impact of community pharmacist involvement on transitions of care: A systematic review and meta-analysis. J Am Pharm Assoc. 2020 Jan;60(1):153–162.e5.	doi:10.1016/j.japh.2019.07.002
71	Ma Z, Dhir P, Perrier L, Bayley M, Munce S. The impact of vocational interventions on vocational outcomes, quality of life, and community integration in adults with childhood onset disabilities: A systematic review. J Occup Rehabil. 2020 Sep;30(1):1–21.	doi:10.1007/s10926-019-09854-1
72	Mac Giolla Phadraig C, Asimakopoulou K, Daly B, Fleischmann I, Nunn J. Nonpharmacological techniques to support patients with intellectual developmental disorders to receive dental treatment: A systematic review of behavior change techniques. Spec Care Dentist. 2020 Nov;40(1):10–25.	doi:10.1111/scd.12434
73	Manohar N, Hayen A, Fahey P, Arora A. Obesity and dental caries in early childhood: A systematic review and meta-analyses. Obes Rev. 2020 Nov;21(3):e12960.	doi:10.1111/obr.12960
74	Martha BA, Vacchi CO, Fattori RA, Macagnan FE. Effect of physical exercise on the functional capacity of children and adolescents submitted to transplantation of hematopoietic stem cells - a systematic review with meta-analysis. J Child Health Care. 2020 Feb;25(1):18–30.	doi:10.1177/1367493520903626
75	Martin J, Viprey M, Castagne B, Merle B, Giroudon C, Chapurlat R, et al. Interventions to improve osteoporosis care: A systematic review and meta-analysis. Osteoporos Int. 2020 Jan;31(3):429–46.	doi:10.1007/s00198-020-05308-0
76	Martson AG, Bakker M, Blokzijl H, Verschuuren EAM, Berger SP, Span LFR, et al. Exploring failure of antimicrobial prophylaxis and pre-emptive therapy for transplant recipients: A systematic review. BMJ Open. 2020 Jan;10(1):e034940.	doi:10.1136/bmjopen-2019-034940
77	Mecenas P, Espinosa DG, Cardoso PC, Normando D. Stainless steel or titanium mini-implants? A systematic review. Angle Orthod. 2020;90(4):587–97.	doi:10.2319/081619-536.1
78	Meuwese CL, Haan M de, Zwetsloot PP, Braithwaite S, Ramjankhan F, Heijden J van der, et al. The hemodynamic effect of different left ventricular unloading techniques during veno-arterial extracorporeal life support: A systematic review and meta-analysis. Perfusion. 2020 Jan;35(7):026765911989747.	doi:10.1177/0267659119897478
79	Mian H, Mian OS, Rochwerg B, Foley R, Wildes TM. Autologous stem cell transplant in older patients (age 65) with newly diagnosed multiple myeloma: A systematic review and meta-analysis. J Geriatr Oncol. 2020 Jan;11(1):93–9.	doi:10.1016/j.jgo.2019.05.014
80	Moda LB, Silva Barros ALC da, Fagundes NCF, Normando D, Maia LC, Anjos Mendes SM dos. Lower fixed retainers: Bonded on all teeth or only on canines? A systematic review. Angle Orthod. 2020 Sep;90(1):125–43.	doi:10.2319/013019-63.1
81	Mujcic A, Blankers M, Bommel J, Boon B, Berman AH, Verdonck-de Leeuw IM, et al. The effectiveness of distance-based interventions for smoking cessation and alcohol moderation among cancer survivors: A meta-analysis. Psychooncology. 2020;29:49–60.	doi:10.1002/pon.5261
82	Muñoz Aguilera E, Suvan J, Buti J, Czesnikiewicz-Guzik M, Barbosa Ribeiro A, Orlandi M, et al. Periodontitis is associated with hypertension: A systematic review and meta-analysis. Cardiovasc Res. 2020 Sep;116(1):28–39.	doi:10.1093/cvr/cvz201
83	Né YGS, Martins BV, Castro MML, Alvarenga MOP, Fagundes NCF, Magno MB, et al. Is nutritional intervention an improvement factor in the management of periodontitis? A systematic review. Clin Nutr. 2020;39(9):2639–46.	doi:10.1016/j.clnu.2019.12.016

	Reference	DOI
84	Nizam MA, Alvi AS, Hamdani MM, Lalani AS, Sibtain SA, Bhangar NA. Efficacy of double versus single phototherapy in treatment of neonatal jaundice: A meta-analysis. Eur J Pediatr. 2020 Jan;179(6):865–74.	doi:10.1007/s00431-020-03583-x
85	Nylander Vujovic S, Nava C, Johansson M, Bruschettini M. Confounding biases in studies on early- versus late-caffeine in preterm infants: A systematic review. Pediatr Res. 2020 Jan;88(3):357–64.	doi:10.1038/s41390-020-0757-1
86	Okolie C, Wood S, Hawton K, Kandalama U, Glendenning AC, Dennis M, et al. Means restriction for the prevention of suicide by jumping. Cochrane Database Syst Rev. 2020 Feb;(2).	doi:10.1002/14651858.CD013543
87	Omori Y, Lang NP, Botticelli D, Papageorgiou SN, Baba S. Biological and mechanical complications of angulated abutments connected to fixed dental prostheses: A systematic review with meta-analysis. J Oral Rehabil. 2020;47:101–11.	doi:10.1111/joor.12877
88	Ordean A, Kim G. Cannabis use during lactation: Literature review and clinical recommendations. J Obstet Gynaecol Can. 2020;42(10):1248–53.	doi:10.1016/j.jogc.2019.11.003
89	Oteri V, Occhipinti F, Gribaudo G, Marastoni F, Chisari E. Integration of ultrasound in medical school: Effects on physical examination skills of undergraduates. Med Sci Educ. 2020 Feb;30(1):417–27.	doi:10.1007/s40670-020-00921-4
90	Palmowski J, Reichel T, Bolau TK, Krger K. The effect of acute running and cycling exercise on T cell apoptosis in humans: A systematic review. Scand J Immunol. 2020 Nov;91(2):e12834.	doi:10.1111/sji.12834
91	Podlasek A, Al Sultan AA, Assis Z, Kashani N, Goyal M, Almekhlafi MA. Outcome of intracranial flow diversion according to the antiplatelet regimen used: A systematic review and meta-analysis. J Neurointerv Surg. 2020;12:148–55.	doi:10.1136/neurintsurg-2019-014996
92	Rayner TA, Harrison S, Rival P, Mahoney DE, Caputo M, Angelini GD, et al. Minimally invasive versus conventional surgery of the ascending aorta and root: A systematic review and meta-analysis. Eur J Cardiothorac Surg. 2020 Jun;57(1):8–17.	doi:10.1093/ejcts/ezz177
93	Rhodes RE, Baranova M, Christian H, Westgarth C. Increasing physical activity by four legs rather than two: Systematic review of dog-facilitated physical activity interventions. Br J Sports Med. 2020 Jan;54(20):1202–7.	doi:10.1136/bjsports-2019-101156
94	Rodrigues BS, David C, Costa J, Ferreira JJ, Pinto FJ, Caldeira D. Influenza vaccination in patients with heart failure: A systematic review and meta-analysis of observational studies. Heart. 2020 Aug;106(5):350–7.	doi:10.1136/heartjnl-2019-315193
95	Rys P, Borys S, Hohendorff J, Zapala A, Witek P, Monica M, et al. NPWT in diabetic foot wounds: A systematic review and meta-analysis of observational studies. Endocrine. 2020;68(1):44–55.	doi:10.1007/s12020-019-02164-9
96	Sagae VMT, Ribeiro IB, Moura DTH de, Brunaldi VO, Logiudice FP, Funari MP, et al. Endoscopic submucosal dissection versus transanal endoscopic surgery for the treatment of early rectal tumor: A systematic review and meta-analysis. Surg Endosc. 2020;34:1025–34.	doi:10.1007/s00464-019-07271-2
97	Schimmack S, Kaiser J, Probst P, Kalkum E, Diener MK, Strobel O. Meta-analysis of α-blockade versus no blockade before adrenalectomy for phaeochromocytoma. Br J Surg. 2020;107(2):e102–8.	doi:10.1002/bjs.11348
98	Schmidt R, Rodrigues CG, Schmidt KH, Irigoyen MCC. Safety and efficacy of baroreflex activation therapy for heart failure with reduced ejection fraction: A rapid systematic review. ESC Heart Fail. 2020 Jan;7(1):3–14.	doi:10.1002/ehf2.12543
99	Schutyser W, Cruyt L, Vulsteke JB, Lenaerts JL, De Langhe E. The role of high-resolution manometry in the assessment of upper gastrointestinal involvement in systemic sclerosis: A systematic review. Clin Rheumatol. 2020;39:149–57.	doi:10.1007/s10067-019-04794-w
100	Sennesael A-L, Krug B, Sneyers B, Spinewine A. Do computerized clinical decision support systems improve the prescribing of oral anticoagulants? A systematic review. Thromb Res. 2020 Mar;187:79–87.	doi:10.1016/j.thromres.2019.12.023
101	Sfakianoudis K, Pantos K, Grigoriadis S, Rapani A, Maziotis E, Tsioulou P, et al. What is the true place of a double stimulation and double oocyte retrieval in the same cycle for patients diagnosed with poor ovarian reserve? A systematic review including a meta-analytical approach. J Assist Reprod Genet. 2020 Dec;37(1):181–204.	doi:10.1007/s10815-019-01638-z

	Reference	DOI
102	Sideris A, Holmes TR, Cumming B, Havas T. A systematic review and meta-analysis of predictors of airway intervention in adult epiglottitis. Laryngoscope. 2020;130:465–73.	doi:10.1002/lary.28076
103	Singh AD, Maitra S, Singh N, Tyagi P, Ashraf A, Kumar R, et al. Systematic review with meta-analysis: Impact of baseline resistance-associated substitutions on the efficacy of glecaprevir/pibrentasvir among chronic hepatitis C patients. Aliment Pharmacol Ther. 2020 Jan;51(5):490–504.	doi:10.1111/apt.15633
104	Sletvold H, Sagmo LAB, Torheim EA. Impact of pictograms on medication adherence: A systematic literature review. Patient Educ Couns. 2020;103(6):1095–103.	doi:10.1016/j.pec.2019.12.018
105	Sterpetti AV, Costi U, D'Ermo G. National statistics about resection of the primary tumor in asymptomatic patients with Stage IV colorectal cancer and unresectable metastases. Need for improvement in data collection. A systematic review with meta-analysis. Surg Oncol. 2020 Jun;33:11–8.	doi:10.1016/j.suronc.2019.12.004
106	Su S-F, Wu M-S, Yeh W-T, Liao Y-C. Effects of lumbar fusion surgery with ISOBAR devices versus posterior lumbar interbody fusion surgery on pain and disability in patients with lumbar degenerative diseases: A meta-analysis. J Invest Surg. 2020;33:79–93.	doi:10.1080/08941939.2018.1472318
107	Suarez S, Conde-Agudelo A, Borovac-Pinheiro A, Suarez-Rebling D, Eckardt M, Theron G, et al. Uterine balloon tamponade for the treatment of postpartum hemorrhage: A systematic review and meta-analysis. Am J Obstet Gynecol. 2020 Apr;222(4):293.e1–52.	doi:10.1016/j.ajog.2019.11.1287
108	Tadount F, Doyon-Plourde P, Rafferty E, MacDonald S, Sadarangani M, Quach C. Is there a difference in the immune response, efficacy, effectiveness and safety of seasonal influenza vaccine in males and females? - a systematic review. Vaccine. 2020 Jan;38(3):444–59.	doi:10.1016/j.vaccine.2019.10.091
109	Tan SM, Han E, Quek RYC, Singh SR, Gea-Snchez M, Legido-Quigley H. A systematic review of community nursing interventions focusing on improving outcomes for individuals exhibiting risk factors of cardiovascular disease. J Adv Nurs. 2020 Oct;76(1):47–61.	doi:10.1111/jan.14218
110	Tlapa D, Zepeda-Lugo CA, Tortorella GL, Baez-Lopez YA, Limon-Romero J, Alvarado-Iniesta A, et al. Effects of lean healthcare on patient flow: A systematic review. Value Health. 2020 Feb;23(2):260–73.	doi:10.1016/j.jval.2019.11.002
111	Triantafyllou T, Olson MT, Theodorou D, Schizas D, Singhal S. Enhanced recovery pathways vs standard care pathways in esophageal cancer surgery: Systematic review and meta-analysis. Esophagus. 2020 Jan;17(2):100–12.	doi:10.1007/s10388-020-00718-9
112	Tsaousi G, Pourzitaki C, Siafis S, Kyrgidis A, Grosomanidis V, Kouvelas D, et al. Levetiracetam as preventive treatment in adults with migraine: An up-to-date systematic review and quantitative meta-analysis. Eur J Clin Pharmacol. 2020 Nov;76(2):161–74.	doi:10.1007/s00228-019-02790-2
113	Tseng M, Vellayappan B, Choong R, Appalanaido GK, Soon YY. Post mastectomy radiotherapy for elderly patients with intermediate risk (T1-2N1 or T3N0) breast cancer: A systematic review and meta-analysis. Transl Cancer Res. 2020 Jan;9(S1):S23–8.	doi:10.21037/tcr.2019.07.23
114	Tzoumas A, Giannopoulos S, Texakalidis P, Charisis N, Machinis T, Koullias GJ. Synchronous versus staged carotid endarterectomy and coronary artery bypass graft for patients with concomitant severe coronary and carotid artery stenosis: A systematic review and meta-analysis. Ann Vasc Surg. 2020 Feb;63:427–438.e1.	doi:10.1016/j.avsg.2019.09.007
115	Veloso A, Vicente SG, Filipe MG. Effectiveness of cognitive training for school-aged children and adolescents with Attention Deficit/Hyperactivity Disorder: A systematic review. Front Psychol. 2020 Jan;10:2983.	doi:10.3389/fpsyg.2019.02983

	Reference	DOI
116	Vygen-Bonnet S, Hellenbrand W, Garbe E, Von Kries R, Bogdan C, Heininger U, et al. Safety and effectiveness of acellular pertussis vaccination during pregnancy: A systematic review. BMC Infect Dis. 2020 Feb;20(1):136.	doi:10.1186/s12879-020-4824-3
117	Wang K-W, Ladhani S, Empringham B, Portwine C, Fleming A, Banfield L, et al. Bariatric interventions in obesity treatment and prevention in pediatric acute lymphoblastic leukemia: A systematic review and meta-analysis. Cancer Metastasis Rev. 2020 Jan;39(1):79–90.	doi:10.1007/s10555-020-09849-y
118	Wiseman-Hakes C, Ryu H, Lightfoot D, Kukreja G, Colantonio A, Matheson FI. Examining the efficacy of communication partner training for improving communication interactions and outcomes for individuals with traumatic brain injury: A systematic review. Arch Rehabil Res Clin Transl. 2020 Mar;2(1):100036.	doi:10.1016/j.arrct.2019.100036
119	Wong CK, O'Rielly CM, Teitge BD, Sutherland RL, Farquharson S, Ghosh M, et al. The characteristics and effectiveness of interventions for frequent emergency department utilizing patients with chronic non-cancer pain: A systematic review. Asher S, editor. Acad Emerg Med. 2020 Mar;27(8):742–52.	doi:10.1111/acem.13934
120	Young I, Waddell LA, Wilhelm BJ, Greig J. A systematic review and meta-regression of single group, pre-post studies evaluating food safety education and training interventions for food handlers. Food Res Int. 2020 Feb;128:108711.	doi:10.1016/j.foodres.2019.108711
121	Zha L, Pan L, Guo J, French N, Villanueva EV, Tefsen B. Effectiveness and safety of high dose tigecycline for the treatment of severe infections: A systematic review and meta-analysis. Adv Ther. 2020 Jan;37(3):1049–64.	doi:10.1007/s12325-020-01235-y
122	Zhao B, Lv W, Mei D, Luo R, Bao S, Huang B, et al. Comparison of short-term surgical outcome between 3D and 2D laparoscopy surgery for gastrointestinal cancer: A systematic review and meta-analysis. Langenbecks Arch Surg. 2020 Jan;405(1):1–2.	doi:10.1007/s00423-020-01853-8
123	Zittermann A, Pilz S, Berthold HK. Serum 25-hydroxyvitamin D response to vitamin D supplementation in infants: A systematic review and meta-analysis of clinical intervention trials. Eur J Nutr. 2020 Feb;59(1):359–69.	doi:10.1007/s00394-019-01912-x
124	Zymperdikas VF, Yavropoulou MP, Kaklamanos EG, Papadopoulos MA. Effects of systematic bisphosphonate use in patients under orthodontic treatment: A systematic review. Eur J Orthod. 2020 Apr;42:60–71.	doi:10.1093/ejo/cjz021

Supplementary Table 2: Publications excluded after full-text screening

	Reference	DOI	Reason for exclusion
1	Agarwal R, Liao JM, Gupta A, Navathe AS. The impact of bundled payment on health care spending, utilization, and quality: A systematic review. Health Aff (Millwood). 2020 Jan;39(1):50–7.	doi:10.1377/hlthaff.2019.00784	Did not use ROBINS-I
2	Albanese E, Bütikofer L, Armijo-Olivo S, Ha C, Egger M. Construct validity of the Physiotherapy Evidence Database (PEDro) quality scale for randomized trials: Item response theory and factor analyses. Res Synth Meth. 2020 Jan;11(2):227–36.	doi:10.1002/jrsm.1385	Not a systematic review
3	Avgerinos KI, Egan JM, Mattson MP, Kapogiannis D. Medium chain triglycerides induce mild ketosis and may improve cognition in Alzheimer's disease. A systematic review and meta-analysis of human studies. Ageing Res Rev. 2020 Mar;58:101001.	doi:10.1016/j.arr.2019.101001	Did not use ROBINS-I
4	Babic A, Vuka I, Saric F, Proloscic I, Slapnicar E, Cavar J, et al. Overall bias methods and their use in sensitivity analysis of Cochrane reviews were not consistent. J Clin Epidemiol. 2020 Mar;119:57–64.	doi:10.1016/j.jclinepi.2019.11.008	Not a systematic review
5	Besomi M, Maclachlan L, Mellor R, Vicenzino B, Hodges PW. Tensor fascia latae muscle structure and activation in individuals with lower limb musculoskeletal conditions: A systematic review and meta-analysis. Sports Med. 2020 Jan;50(5):965–85.	doi:10.1007/s40279-019-01251-1	Did not use ROBINS-I
6	Blakeway H, Van-de-Velde V, Allen VB, Kravvas G, Palla L, Page MJ, et al. What is the evidence for interactions between filaggrin null mutations and environmental exposures in the aetiology of atopic dermatitis? A systematic review. Br J Dermatol. 2020 Feb;183(3):443–51.	doi:10.1111/bjd.18778	Did not use ROBINS-I
7	Brauers L, Rameckers E, Severijns D, Feys P, Smeets R, Klingels K. Measuring motor fatigability in the upper limbs in individuals with neurologic disorders: A systematic review. Arch Phys Med Rehabil. 2020 May;101(5):907–16.	doi:10.1016/j.apmr.2019.11.015	No full text available
8	Cao S, Jones M, Tooth L, Mishra GD. History of premenstrual syndrome and development of postpartum depression: A systematic review and meta-analysis. J Psychiatr Res. 2020 Feb;121:82–90.	doi:10.1016/j.jpsychires.2019.11.010	Did not use ROBINS-I
9	Cohen JA, Trojano M, Mowry EM, Uitdehaag BMJ, Reingold SC, Marrie RA. Leveraging real-world data to investigate multiple sclerosis disease behavior, prognosis, and treatment. Mult Scler J. 2020 Nov;26(1):23–37.	doi:10.1177/1352458519892555	Not a systematic review
10	Dhiman P, Lee H, Kirtley S, Collins GS. A systematic review showed more consideration is needed when conducting nonrandomized studies of interventions. J Clin Epidemiol. 2020 Jan;117:99–108.	doi:10.1016/j.jclinepi.2019.09.027	Methodological
11	Dimitropoulos K, Gravas S. Mind the gap: Management of benign prostatic obstruction (BPO) surgical candidates on antithrombotics. World J Urol. 2020;38:247–8.	doi:10.1007/s00345-018-02625-2	Not a systematic review
12	Ewald H, Ioannidis JPA, Ladanie A, Mc Cord K, Bucher HC, Hemkens LG. Nonrandomized studies using causal-modeling may give different answers than RCTs: A meta-epidemiological study. J Clin Epidemiol. 2020 Feb;118:29–41.	doi:10.1016/j.jclinepi.2019.10.012	Did not use ROBINS-I
13	Franklin JM, Glynn RJ, Suissa S, Schneeweiss S. Emulation differences vs. Biases when calibrating real-world evidence findings against randomized controlled trials. Clin Pharmacol Ther. 2020 Feb;107(4):735–7.	doi:10.1002/cpt.1793	Not a systematic review

	Reference	DOI	Reason for exclusion
14	Haby MM, Sosa Leon LA, Lucianez A, Nicholls RS, Reveiz L, Donadeu M. Systematic review of the effectiveness of selected drugs for preventive chemotherapy for Taenia solium taeniasis. Ozkan AT, editor. PLoS NeglTrop Dis. 2020 Jan;14(1):e0007873.	doi:10.1371/journal.pntd.0007873	Did not use ROBINS-I
15	Howden CW, Korvick JA, Harinstein L, Weissfeld J, Moayyedi P, Vakil N, et al. Controversies around measuring drug toxicity: US Food and Drug Administration and gastrointestinal perspectives. Gastroenterology. 2020 Jan;158(1):22–7.	doi:10.1053/j.gastro.2019.10.014	Not a systematic review
16	Huddart S, Svadzian A, Nafade V, Satyanarayana S, Pai M. Tuberculosis case fatality in India: A systematic review and meta-analysis. BMJ Glob Health. 2020;5(1):e002080.	doi:10.1136/bmjgh-2019-002080	Did not use ROBINS-I
17	Hughes SL, Bolotin S, Khan S, Li Y, Johnson C, Friedman L, et al. The effect of time since measles vaccination and age at first dose on measles vaccine effectiveness - a systematic review. Vaccine. 2020;38:460–9.	doi:10.1016/j.vaccine.2019.10.090	Did not use ROBINS-I
18	Jang M-A, Kim B, Lee YK. Reporting quality of diagnostic accuracy studies in laboratory medicine: Adherence to standards for reporting of diagnostic accuracy studies (STARD) 2015. Ann Lab Med. 2020;40:245–52.	doi:10.3343/alm.2020.40.3.274	Did not use ROBINS-I
19	Lazzara EH, Keebler JR, Simonson RJ, Agarwala A, Lane-Fall MB. Navigating the challenges of performing anesthesia handoffs and conducting anesthesia handoff research. Int Anesthesiol Clin. 2020 Nov;58(1):32–7.	doi:10.1097/AIA.0000000000000260	Not a systematic review
20	Lee DJ, Yang W, Propst EJ, Rosenblatt SD, Hseu A, Wolter NE. Tracheo-innominate fistula in children: A systematic review of literature. Laryngoscope. 2020;130:217–24.	doi:10.1002/lary.27765	Did not use ROBINS-I
21	Manerkar K, Harding J, Conlon C, McKinlay C. Maternal gestational diabetes and infant feeding, nutrition and growth: A systematic review and meta-analysis. Br J Nutr. 2020 Jan;123(11):1201–15.	doi:10.1017/S0007114520000264	Did not use ROBINS-I
22	McNeil D, Provencher M, Wong IH. Arthroscopic anatomic glenoid reconstruction demonstrates its safety with short-term to medium-term results for anteroinferior shoulder instability: A systematic review. J ISAKOS. 2020;5:37–47.	doi:10.1136/jisakos-2019-000388	No full text available
23	Moskalewicz A, Oremus M. No clear choice between Newcastle-Ottawa Scale and appraisal tool for cross-sectional studies to assess methodological quality in cross-sectional studies of health-related quality of life and breast cancer. J Clin Epidemiol. 2020 Apr;120:103–94.	doi:10.1016/j.jclinepi.2019.12.013	Did not use ROBINS-I
24	Noly P-E, Ben Ali W, Lamarche Y, Carrier M. Status, indications, and use of cardiac replacement therapy in the era of multimodal mechanical approaches to circulatory support: A scoping review. Can J Cardiol. 2020;36:261–9.	doi:10.1016/j.cjca.2019.11.027	Not a systematic review
25	Puhl RM. What words should we use to talk about weight? A systematic review of quantitative and qualitative studies examining preferences for weight-related terminology. Obes Rev. 2020 Feb;21(6):e13008.	doi:10.1111/obr.13008	Did not use ROBINS-I
26	Radke EG, Braun JM, Nachman RM, Cooper GS. Phthalate exposure and neurodevelopment: A systematic review and meta-analysis of human epidemiological evidence. Environ Int. 2020;137:105408.	doi:10.1016/j.envint.2019.105408	Did not use ROBINS-I
27	Sacco R, Shah S, Leeson R, Moraschini V, Almeida Barros Mouro CF de, Akintola O, et al. Osteonecrosis and osteomyelitis of the jaw associated with tumour necrosis factor-alpha (TNF-α) inhibitors: A systematic review. Br J Oral Maxillofac Surg. 2020;58:25–33.	doi:10.1016/j.bjoms.2019.09.023	Did not use ROBINS-I

	Reference	DOI	Reason for exclusion
28	Schardong J, Stein C, Della Mea Plentz R. Neuromuscular electrical stimulation in chronic kidney failure: A systematic review and meta-analysis. Arch Phys Med Rehabil. 2020;101(4):700–11.	doi:10.1016/j.apmr.2019.11.008	No full text available
29	Thaivalappil A, Young I, Paco C, Jeyapalan A, Papadopoulos A. Food safety and the older consumer: A systematic review and meta-regression of their knowledge and practices at home. Food Control. 2020 Jan;107:106782.	doi:10.1016/j.foodcont.2019.106782	Did not use ROBINS-I
30	Tocalini P, Vicente A, Amoza RL, Garca Reid C, Cura AJ, Tozzi WA, et al. Association between obesity and mortality in adult patients receiving invasive mechanical ventilation: A systematic review and meta-analysis. Med Intensiva. 2020 Jan;44(1):18–26.	doi:10.1016/j.medin.2018.07.006	Did not use ROBINS-I
31	Van Grootven B, Mendelson DA, Deschodt M. Impact of geriatric co-management programmes on outcomes in older surgical patients: Update of recent evidence. Curr Opin Anaesthesiol. 2020 Feb;33(1):114–21.	doi:10.1097/ACO.0000000000000815	Not a systematic review
32	Wu M-Y, Lo W-C, Chao C-T, Wu M-S, Chiang C-K. Association between air pollutants and development of chronic kidney disease: A systematic review and meta-analysis. Sci Total Environ. 2020 Mar;706:135522.	doi:10.1016/j.scitotenv.2019.135522	Did not use ROBINS-I
33	Zuckier LS. Evidence-based medicine in the domain of nuclear medicine, the fifty-year view. Semin Nucl Med. 2020 Jan;50(1):110–4.	doi:10.1053/j.semnuclmed.2019.07.007	Not a systematic review

Supplementary Table 3: Characteristics of the included systematic reviews

	First author	Type of intervention	Included studies			Extracted RoB ratings	MA?	Funding	Competing interests	AMSTAR 2 confidence rating
			NRS	RCT	Total					
1	Agnelli	Non-interventional	105	19	124	107	Yes	Industry	Industry	Critically low
2	Alshafei	Clinical intervention (surgical)	4	2	6	4	Yes	None	None	Critically low
3	Arn	Clinical intervention (surgical)	18	11	29	5	No	Not reported	None	Critically low
4	Baker	Clinical intervention (other)	11	19	30		No	None	None	Critically low
5	Balla	Clinical intervention (surgical)	28		28	28	No	Not reported	None	Critically low
6	Bang	Clinical intervention (surgical)	8		8	8	Yes	Government	None	Critically low
7	Belachew	Public health intervention	11		11		Yes	Not reported	None	Critically low
8	Belk	Clinical intervention (surgical)	11	1	12	12	Yes	Not reported	None	Critically low
9	Bellos	Non-interventional	17		17	17	Yes	None	None	Low
10	Bezeczky	Non-clinical intervention	14	15	29	13	Yes	Government	Academic	Low
11	Brennan	Environmental exposure	27		27	18	Yes	Government	None	High
12	Brouwer	Clinical intervention (other)	4	7	11	4	Yes	Not reported	None	Critically low
13	Cardoso	Clinical intervention (surgical)	6	1	7		No	None	None	High
14	Carrier	Clinical intervention (other)	29	7	36	29	Yes	Foundation/NGO	None	Moderate
15	Carter	Clinical intervention (other)	24		24	24	No	Multiple (excl. industry)	Academic	Low
16	Checcucci (a)	Clinical intervention (surgical)	10		10	7	No	None	None	Critically low
17	Checcucci (b)	Clinical intervention (surgical)	5	3	8		Yes	Not reported	None	Critically low
18	Chen	Clinical intervention (drug)	7	5	12	7	Yes	None	None	Critically low
19	Coates	Non-interventional	62	6	68	46	No	Multiple (excl. industry)	None	Moderate
20	Cutts	Non-interventional	28	5	33	16	Yes	Government	None	Critically low
21	Daugaard	Clinical intervention (other)	5	9	14	9	No	Multiple (excl. industry)	Industry	Low
22	Davidson	Clinical intervention (other)	27		27	27	No	Not reported	None	Low
23	DeNicola	Public health intervention	8	39	47		No	Multiple (incl. industry)	Industry	Low
24	Di Giuseppe	Clinical intervention (other)	24		24	24	No	Not reported	None	Critically low
25	Djade	Non-interventional	6		6	6	No	Not reported	Not reported	Critically low
26	Djarv	Clinical intervention (drug)	2	1	3		Yes	Foundation/NGO	None	Critically low
27	Falk Brekke	Clinical intervention (other)	2	2	4	2	No	None	Industry	Low
28	Fang	Non-interventional	12		12	12	Yes	Government	None	Moderate
29	Ford	Clinical intervention (other)	14	2	16	2	No	None	None	Moderate
30	Frascella	Public health intervention	2	9	11		No	Industry	None	Low
31	Froggatt	Environmental exposure	22		22		Yes	Government	None	Low
32	Gagelmann	Clinical intervention (other)	18		18		Yes	Not reported	None	Critically low
33	Gandolla	Clinical intervention (other)	14		14	14	Yes	Foundation/NGO	None	Critically low
34	Garbazza	Non-interventional	39	1	40	4	Yes	Government	None	Critically low

MA: Meta-analysis. NRS: Non-randomised study. RCT: Randomised controlled trial. RoB: Risk of bias.

	First author	Type of intervention	Included studies NRS	RCT	Total	Extracted RoB ratings	MA?	Funding	Competing interests	AMSTAR 2 confidence rating
35	Geierlehner	Clinical intervention (surgical)	32		32	7	Yes	Government	None	Low
36	Gentile	Non-interventional	14		14		Yes	None	None	Critically low
37	Giannopoulos	Clinical intervention (surgical)	5		5	5	Yes	None	None	Critically low
38	Giles	Clinical intervention (surgical)	18		18		Yes	None	None	Critically low
39	Gogou	Clinical intervention (drug)	13	6	19		No	Not reported	None	Critically low
40	Goldshtein	Public health intervention	9		9		No	None	None	Low
41	Gottlieb	Clinical intervention (drug)	3		3	3	Yes	None	None	Moderate
42	Graham	Non-interventional	21		21		Yes	Multiple (excl. industry)	None	Moderate
43	Guo	Clinical intervention (drug)	13	1	14	11	Yes	None	None	Critically low
44	Gupta	Clinical intervention (other)	10	2	12	10	Yes	Industry	Industry	Critically low
45	Hajibandeh	Clinical intervention (surgical)	5	4	9		Yes	None	None	Critically low
46	Hajibandeh	Clinical intervention (surgical)	11		11		Yes	Not reported	None	Low
47	Handel	Environmental exposure	29		29	29	Yes	Industry	Industry	High
48	Hasperhoven	Public health intervention	17		17	17	Yes	None	Academic	Low
49	Heesen	Clinical intervention (other)	12	1	13	12	Yes	None	None	Low
50	Hsu	Clinical intervention (drug)	4	8	12	4	Yes	Academic	None	Critically low
51	Hu	Clinical intervention (other)	14	5	19		No	Not reported	None	Moderate
52	Huang	Clinical intervention (other)	7	11	18	7	Yes	Multiple (excl. industry)	None	Critically low
53	Indarwati	Non-interventional	26	6	32	7	Yes	None	Industry	Critically low
54	Jassal	Clinical intervention (drug)	34	4	38	35	No	None	None	Moderate
55	Kamfose	Clinical intervention (drug)	4	2	6	4	No	Government	None	Low
56	Karamini	Clinical intervention (other)	1	3	4	1	No	Not reported	None	Critically low
57	Kling	Clinical intervention (drug)	17	15	32	25	No	None	Academic	Low
58	Koenig	Clinical intervention (drug)	13		13	13	Yes	Government	None	Critically low
59	Kokkinidis	Clinical intervention (drug)	19		19		Yes	None	Industry	Low
60	Kolding	Clinical intervention (drug)	25	7	32	27	No	None	None	Low
61	Kowalewski	Public health intervention	54		54	54	Yes	None	None	Critically low
62	Lam	Non-interventional	16		16		Yes	None	None	High
63	Leach	Public health intervention	1	1	2		No	None	None	Moderate
64	Lee (a)	Public health intervention	1	2	3	1	No	Government	Academic	High
65	Lee (b)	Public health intervention	13	1	14	13	Yes	Other	None	Low
66	Lee (c)	Clinical intervention (surgical)	13		13		No	None	None	Critically low
67	Liu	Public health intervention	33	4	37	33	No	Government	None	Low
68	Louwers	Clinical intervention (surgical)	12		12	12	No	Not reported	Not reported	Low
69	Lu	Clinical intervention (surgical)	5		5	5	Yes	Government	None	Critically low
70	Lussier	Public health intervention	13	23	36	6	Yes	Foundation/NGO	Academic	Low

MA: Meta-analysis. NRS: Non-randomised study. RCT: Randomised controlled trial. RoB: Risk of bias.

	First author	Type of intervention	Included studies NRS	RCT	Total	Extracted RoB ratings	MA?	Funding	Competing interests	AMSTAR 2 confidence rating
71	Ma	Non-clinical intervention	14	3	17	14	No	Multiple (excl. industry)	None	Moderate
72	Mac Giolla Phadraig	Clinical intervention (other)	22	1	23	10	No	None	None	Moderate
73	Manohar	Non-interventional	9		9	9	Yes	Multiple (excl. industry)	None	High
74	Martha	Clinical intervention (other)	2	1	3	2	Yes	None	None	Critically low
75	Martin	Public health intervention	14	18	32	14	Yes	Foundation/NGO	Industry	Critically low
76	Martson	Clinical intervention (drug)	8	16	24	8	No	Foundation/NGO	None	Low
77	Mecenas	Clinical intervention (surgical)	5	1	6		No	Not reported	Not reported	Moderate
78	Meuwese	Clinical intervention (surgical)	8		8	7	Yes	None	Industry	Critically low
79	Mian	Clinical intervention (other)	6	2	8	6	Yes	None	Industry	Moderate
80	Moda	Clinical intervention (surgical)	3	2	5	3	No	Not reported	Not reported	Low
81	Mujcic	Public health intervention	5	12	17	1	Yes	Foundation/NGO	Academic	Critically low
82	Muñoz Aguilera	Non-interventional	74	8	82	2	Yes	Government	None	Critically low
83	Né	Clinical intervention (other)	3	1	4	3	No	None	None	Moderate
84	Nizam	Clinical intervention (other)	4	6	10	4	Yes	Not reported	None	Critically low
85	Nylander Vujovic	Clinical intervention (drug)	14	2	16	13	No	Government	None	Moderate
86	Okolie	Public health intervention	14		14	13	Yes	Multiple (excl. industry)	Academic	High
87	Omori	Clinical intervention (surgical)	9		9	9	Yes	Multiple (incl. industry)	None	Low
88	Ordean	Environmental exposure	2		2	2	No	Academic	Academic	Critically low
89	Oteri	Public health intervention	15		15		No	None	None	Low
90	Palmowski	Clinical intervention (other)	5	1	6		No	Not reported	Academic	Moderate
91	Podlasek	Clinical intervention (drug)	54		54	5	Yes	None	None	Critically low
92	Rayner	Clinical intervention (surgical)	13		13	15	Yes	Multiple (excl. industry)	None	Low
93	Rhodes	Public health intervention	5	8	13		No	Multiple (excl. industry)	None	Critically low
94	Rodrigues	Clinical intervention (drug)	8		8	10	Yes	None	Industry	Low
95	Rys	Clinical intervention (other)	16		16		Yes	Government	None	Low
96	Sagae	Clinical intervention (surgical)	6		6	6	Yes	None	Industry	Low
97	Schimmack	Clinical intervention (drug)	4		4	4	Yes	None	None	Low
98	Schmidt	Clinical intervention (other)	9	1	10	1	No	Not reported	Not reported	Low
99	Schutyser	Clinical intervention (drug)	15		15	2	No	Foundation/NGO	None	Low
100	Sennesael	Public health intervention	16		16		No	Government	None	Critically low
101	Sfakianoudis	Clinical intervention (drug)	9		9	14	Yes	Not reported	None	Critically low
102	Sideris	Non-interventional	30		30	30	Yes	None	None	Critically low
103	Singh	Clinical intervention (drug)	15	2	17	10	Yes	Government	None	Low
104	Sletvold	Public health intervention	5	18	23	5	No	Multiple (excl. industry)	Industry	Critically low
105	Sterpetti	Clinical intervention (surgical)	27		27	27	Yes	Not reported	None	Critically low
106	Su	Clinical intervention (surgical)	15	3	18		Yes	Not reported	None	Critically low

MA: Meta-analysis. NRS: Non-randomised study. RCT: Randomised controlled trial. RoB: Risk of bias.

	First author	Type of intervention	Included studies NRS	RCT	Total	Extracted RoB ratings	MA?	Funding	Competing interests	AMSTAR 2 confidence rating
107	Suarez	Clinical intervention (other)	84	7	91	15	Yes	None	Academic	Moderate
108	Tadount	Clinical intervention (drug)	15	31	46	28	No	Government	None	Moderate
109	Tan	Public health intervention	26	20	46		No	Government	None	Low
110	Tlapa	Public health intervention	40		40	40	No	None	None	Low
111	Triantafyllou	Clinical intervention (other)	4	4	8	4	Yes	Not reported	None	Critically low
112	Tsaousi	Clinical intervention (drug)	6	3	9	6	Yes	None	None	Critically low
113	Tseng	Clinical intervention (other)	2		2	4	Yes	Not reported	None	Critically low
114	Tzoumas	Clinical intervention (surgical)	11		11	11	Yes	None	None	Critically low
115	Veloso	Clinical intervention (other)	4	18	22	4	No	Government	None	Critically low
116	Vygen-Bonnet	Clinical intervention (drug)	19	3	22	19	No	None	Industry	Low
117	Wang	Clinical intervention (other)	6	4	10	1	Yes	Not reported	None	Low
118	Wiseman-Hakes	Clinical intervention (other)	9	3	12	3	No	Government	None	Moderate
119	Wong	Public health intervention	9	4	13		No	Foundation/NGO	None	Low
120	Young	Non-clinical intervention	85		85	137	Yes	Not reported	Not reported	Moderate
121	Zha	Clinical intervention (drug)	9	1	10	9	Yes	Other	None	Low
122	Zhao	Clinical intervention (surgical)	11	1	12		Yes	Government	None	Critically low
123	Zittermann	Clinical intervention (drug)	27		27	27	Yes	None	None	Low
124	Zymperdikas	Clinical intervention (drug)	7		7	1	No	None	None	Moderate

MA: Meta-analysis. NRS: Non-randomised study. RCT: Randomised controlled trial. RoB: Risk of bias.

Supplementary Table 4: Modifications and non-standard uses of ROBINS-I

	First author	Reported duplicate ROBINS-I assessment	Reported ROBINS-I pre-assessment stage	Reported overall judgements	Reported domain-specific judgements	Reported justifications for RoB judgements	Rating scale	Other issues in the application of ROBINS-I
1	Agnelli	Yes	None	Yes	Yes	None	Standard scale	Omitted domain 4
2	Alshafei	Yes	None	Yes	Partially	None	Standard scale	
3	Arn	No	None	Yes	Yes	None	Standard scale	
4	Baker	Yes	None	Yes	No	None	Low, Moderate, High	
5	Balla	Yes	None	Yes	Yes	None	Standard scale	
6	Bang	Yes	None	Yes	Yes	None	Standard scale	
7	Belachew	Yes	None	No	No	None	None	
8	Belk	Yes	None	No	Yes	None	Standard scale	
9	Bellos	Yes	None	Yes	Yes	None	Standard scale	
10	Bezeczky	Yes	None	Yes	No	None	Standard scale	
11	Brennan	Partially	Confounders and co-interventions listed	Yes	Yes	Overall and bias domains	Standard scale	
12	Brouwer	Yes	None	Yes	Yes	None	Standard scale	
13	Cardoso	No	None	Yes	Yes	None	Low, Moderate, High	
14	Carrier	Yes	Confounders and co-interventions listed	Yes	Yes	None	Standard scale	Included critical-RoB studies in synthesis.
15	Carter	Yes	None	Yes	Yes	Bias domains only	Standard scale	
16	Checcucci (a)	No	None	No	Yes	None	Standard scale	Reported domains 3 and 4 as ‘Measurement of Exposure’ and ‘Departure from Exposure’.
17	Checcucci (b)	Yes	None	Yes	Partially	None	Low, Moderate, High	
18	Chen	Yes	None	Yes	Yes	None	Standard scale	
19	Coates	Yes	None	Yes	No	None	Standard scale	
20	Cutts	No	None	No	Yes	None	Standard scale	
21	Daugaard	No	Confounders listed	Yes	Yes	None	Standard scale	Included critical-RoB studies in synthesis.
22	Davidson	No	None	Yes	Yes	None	Standard scale	Included critical-RoB studies in synthesis.

RoB: Risk of bias.

	First author	Reported duplicate ROBINS-I assessment	Reported ROBINS-I pre-assessment stage	Reported overall judgements	Reported domain-specific judgements	Reported justifications for RoB judgements	Rating scale	Other issues in the application of ROBINS-I
23	DeNicola	Yes	None	Yes	Partially	None	Low, High, Unclear	
24	Di Giuseppe	Yes	None	Yes	Yes	None	Standard scale	Included critical-RoB studies in synthesis.
25	Djade	Yes	None	No	Yes	Bias domains only	Standard scale	
26	Djarv	Yes	None	No	Partially	None	Not serious, Serious	
27	Falk Brekke	Yes	None	Yes	Yes	None	Standard scale	Included critical-RoB studies in synthesis.
28	Fang	Yes	Confounders listed	Yes	Yes	None	Standard scale	
29	Ford	Yes	None	Yes	Yes	None	Standard scale	
30	Frascella	No	None	Yes	Yes	None	Green, Yellow, Red	
31	Froggatt	No	None	Yes	Yes	None	Low, Moderate, High	
32	Gagelmann	No	None	Yes	Yes	None	Yes, Probably Yes, Probably No, No	Included critical-RoB studies in meta-analysis.
33	Gandolla	Yes	None	No	Yes	Bias domains only	Standard scale	
34	Garbazza	No	None	No	Yes	None	Standard scale	
35	Geierlehner	No	None	Yes	Yes	None	Standard scale	
36	Gentile	No	None	No	No	None	None	
37	Giannopoulos	Yes	None	Yes	Yes	None	Standard scale	
38	Giles	Yes	None	Yes	Yes	None	+, -, Not Assessable	
39	Gogou	Yes	None	Yes	Yes	None	Low, Moderate, Serious, Unknown	Reported domain 3 as ‘Not applicable’ for all studies.
40	Goldshtein	Yes	None	Yes	No	Overall only	Low, Moderate, Serious	
41	Gottlieb	Yes	None	Yes	Yes	None	Standard scale	
42	Graham	Yes	Confounders listed	No	No	None	Standard scale	
43	Guo	Yes	None	Yes	Yes	None	Standard scale	
44	Gupta	Yes	None	Yes	Yes	None	Standard scale	
45	Hajibandeh	Yes	None	No	Yes	None	Low, High, Unclear	
46	Hajibandeh	Yes	None	No	Yes	None	Low, High, Unclear	
47	Handel	No	None	Yes	Yes	None	Standard scale	

RoB: Risk of bias.

	First author	Reported duplicate ROBINS-I assessment	Reported ROBINS-I pre-assessment stage	Reported overall judgements	Reported domain-specific judgements	Reported justifications for RoB judgements	Rating scale	Other issues in the application of ROBINS-I
48	Hasperhoven	No	None	Yes	Yes	None	Standard scale	Included critical-RoB studies in meta-analysis.
49	Heesen	Yes	None	No	Yes	None	Standard scale	
50	Hsu	Yes	None	Yes	Yes	None	Standard scale	
51	Hu	No	None	No	Yes	None	High Quality, Low Quality	Non-standard method for determining overall judgement.[1]
52	Huang	Yes	None	Yes	Partially	None	Standard scale	Included critical-RoB studies in meta-analysis.
53	Indarwati	Yes	None	Yes	Yes	None	Standard scale	
54	Jassal	No	None	No	Yes	None	Standard scale	
55	Kamfose	Yes	None	Yes	Yes	None	Standard scale	Included critical-RoB studies in synthesis.
56	Karamini	Yes	None	Yes	Yes	None	Standard scale	
57	Kling	No	None	Yes	Yes	None	Standard scale	
58	Koenig	Partially	None	Yes	Yes	None	Standard scale	
59	Kokkinidis	Yes	None	No	No	None	None	
60	Kolding	Yes	None	No	Yes	None	Standard scale	
61	Kowalewski	Yes	None	Yes	Yes	None	Standard scale	Included critical-RoB studies in meta-analysis.
62	Lam	No	None	Yes	Yes	None	+, -, ?	
63	Leach	Yes	None	Yes	Yes	None	Low, Moderate, High, Unclear	
64	Lee (a)	Yes	None	Yes	Yes	None	Standard scale	Included critical-RoB studies in synthesis.
65	Lee (b)	Yes	None	Partially	Yes	None	Standard scale	
66	Lee (c)	No	None	Yes	Yes	None	Yes, Probably Yes, Probably No, No, No information	
67	Liu	Yes	None	Yes	Yes	Overall and bias domains	Standard scale	
68	Louwers	Yes	None	Yes	Yes	None	Standard scale	Included critical-RoB studies in synthesis.
69	Lu	Yes	None	Yes	Yes	None	Standard scale	
70	Lussier	No	None	Yes	Yes	None	Standard scale	
71	Ma	Yes	None	Yes	Yes	None	Standard scale	
72	Mac Giolla Phadraig	Yes	None	Yes	Yes	None	Low, Moderate, Serious, Critical, Severe	Included critical-RoB studies in synthesis.
73	Manohar	Yes	None	Yes	Yes	None	Standard scale	Included critical-RoB studies in meta-analysis.
74	Martha	Yes	None	Yes	Yes	None	Standard scale	
75	Martin	Yes	None	Yes	Yes	None	Standard scale	
76	Martson	Yes	None	Yes	Yes	None	Standard scale	Included critical-RoB studies in synthesis.
77	Mecenas	Yes	None	Yes	Yes	None	Low, Moderate, High	

RoB: Risk of bias.

	First author	Reported duplicate ROBINS-I assessment	Reported ROBINS-I pre-assessment stage	Reported overall judgements	Reported domain-specific judgements	Reported justifications for RoB judgements	Rating scale	Other issues in the application of ROBINS-I
78	Meuwese	No	None	No	Yes	None	Standard scale	
79	Mian	Yes	None	Yes	Yes	None	Standard scale	
80	Moda	No	None	Yes	Yes	None	Standard scale	
81	Mujcic	Yes	None	Yes	Yes	None	Standard scale	
82	Muñoz Aguilera	Yes	None	Yes	Yes	None	Standard scale	
83	Né	Yes	None	Yes	Yes	None	Standard scale	
84	Nizam	No	None	Yes	Yes	None	Standard scale	
85	Nylander Vujovic	Yes	Confounders listed	Yes	Yes	None	Standard scale	Included critical-RoB studies in synthesis.
86	Okolie	Yes	None	Yes	Yes	Overall and bias domains	Standard scale	
87	Omori	Yes	None	Yes	Yes	Overall and bias domains	Standard scale	
88	Ordean	No	None	No	Yes	None	Standard scale	
89	Oteri	Yes	None	Yes	Yes	None	Low, Moderate, High	Omitted domain 4
90	Palmowski	Yes	None	No	Yes	None	Low, High, Unclear	Omitted domain 4
91	Podlasek	No	None	Yes	No	None	Standard scale	
92	Rayner	No	None	Yes	Yes	None	Standard scale	
93	Rhodes	Yes	None	Yes	Yes	None	Low, Some Concerns, High	
94	Rodrigues	No	Confounders and co-interventions listed	Yes	Yes	None	Standard scale	
95	Rys	No	None	No	No	None	None	
96	Sagae	No	None	Yes	Yes	None	Standard scale	
97	Schimmack	No	None	Yes	Yes	Overall only	Standard scale	
98	Schmidt	No	None	Yes	Partially	None	Standard scale	
99	Schutyser	Yes	None	Yes	Yes	None	Standard scale	
100	Sennesael	Yes	None	Yes	Yes	None	Low, High, Unclear	Reported ROBINS-I alongside Cochrane RoB 2.0, with domains renamed.[2]
101	Sfakianoudis	Yes	None	Yes	Yes	None	Standard scale	
102	Sideris	No	None	Yes	Yes	None	Standard scale	
103	Singh	Yes	None	Yes	Yes	None	Standard scale	Added domain: ‘Vested interest bias’.[3]
104	Sletvold	Yes	None	Yes	No	None	Standard scale	
105	Sterpetti	Yes	None	Partially	No	None	Standard scale	

RoB: Risk of bias.

	First author	Reported duplicate ROBINS-I assessment	Reported ROBINS-I pre-assessment stage	Reported overall judgements	Reported domain-specific judgements	Reported justifications for RoB judgements	Rating scale	Other issues in the application of ROBINS-I
106	Su	Yes	None	No	No	Bias domains only	None	Only reported free-text descriptions of judgements in each bias domain.[4]
107	Suarez	Yes	None	Yes	Yes	None	Standard scale	Included critical-RoB studies in meta-analysis.
108	Tadount	Yes	Confounders listed	Yes	Partially	None	Standard scale	Included critical-RoB studies in synthesis.
109	Tan	Yes	None	Yes	No	Overall only	Low, Moderate, High	Non-standard process for determining overall score.[5]
110	Tlapa	Yes	None	Yes	No	None	Standard scale	Included critical-RoB studies in synthesis.
111	Triantafyllou	No	None	No	Yes	None	Standard scale	
112	Tsaousi	Yes	None	Yes	Yes	None	Standard scale	Included critical-RoB studies in meta-analysis.
113	Tseng	No	None	Yes	Yes	None	Standard scale	
114	Tzoumas	Yes	None	No	Yes	None	Standard scale	
115	Veloso	Yes	None	Yes	Yes	None	Standard scale	
116	Vygen-Bonnet	Yes	None	Yes	Yes	None	Standard scale	Included critical-RoB studies in synthesis.
117	Wang	No	None	Yes	Yes	Overall and bias domains	Standard scale	
118	Wiseman-Hakes	Yes	None	Yes	Yes	None	Standard scale	
119	Wong	Yes	None	Yes	Partially	None	Low, Moderate, High, N/A	
120	Young	Yes	None	Yes	Yes	Overall and bias domains	Standard scale	Included critical-RoB studies in meta-analysis.
121	Zha	No	None	Yes	Yes	None	Standard scale	Included critical-RoB studies in meta-analysis.
122	Zhao	Yes	None	Yes	Yes	None	Low, Moderate, High	
123	Zittermann	No	None	No	Yes	Bias domains only	Standard scale	
124	Zymperdikas	Yes	None	Yes	Yes	None	Standard scale	Included critical-RoB studies in synthesis.

RoB: Risk of bias.

[1]Quotation: 'Each item in the assessment criteria was given a score of 1 if it was fulfilled (a negative item is considered fulfilled if it is avoided) in the article, and a score of 0 if was not fulfilled or if there was insufficient evidence to make a clear statement. Where a criterion was not considered by the study, it was marked as not applicable. The total score of each study was calculated by dividing the number of items included by the number of applicable items, yielding a score between 0 and 1. The methodological quality was considered low if the score was between 0 and 0.5 and high if the score was between 0.51 and 1.'

[2]Names used for bias domains: 'Confounding', 'Selection bias', 'Performance bias', 'Misclassification bias', 'Attrition bias', 'Detection bias', 'Reporting bias'.

[3]Quotation: 'Vested interest bias was defined as studies that were sponsored by the drug manufacturing pharmaceutical company. Studies were considered to be at a high risk of bias if the sponsoring companies had access to the data and/or were involved in the reporting of study.'

[4]Example of free-text risk-of-bias judgements:

'Confounding: No.

Selection bias: Not described.

Classification of interventions: Clearly defined.

Deviations from intended interventions: No.

Missing data: No loss to follow-up.

Bias in measurement of outcomes: No.

Bias in selection of the reported result: No.'

[5]Quotation: 'Studies that had "low" or "high" risk in most of the domains were classified as having an overall "low" or "high" bias, respectively, while those that yielded "unclear" or "moderate" risks in two or more domains were classified as having an overall "moderate" risk.'

Supplementary Table 5: Overall ROBINS-I risk-of-bias judgements compared to the highest judgement in an individual domain

	Highest individual bias domain judgement								
Overall risk-of-bias judgement	Low		Moderate		Serious		Critical		Total
Low	42%	(30)	28%	(20)	3%	(2)	27%	(19)	71
Moderate	0%	(1)	83%	(276)	13%	(43)	4%	(13)	333
Serious	0%	(0)	1%	(2)	97%	(279)	2%	(6)	287
Critical	0%	(0)	0%	(0)	2%	(4)	98%	(259)	263
No Information	20%	(1)	20%	(1)	60%	(3)	0%	(0)	5

Excludes assessments where any bias domain is missing (including overall) and assessments reported using a non-standard scale.

Supplementary Figure 1: Summary of quality ratings of the included systematic reviews for each individual item in the AMSTAR 2 tool

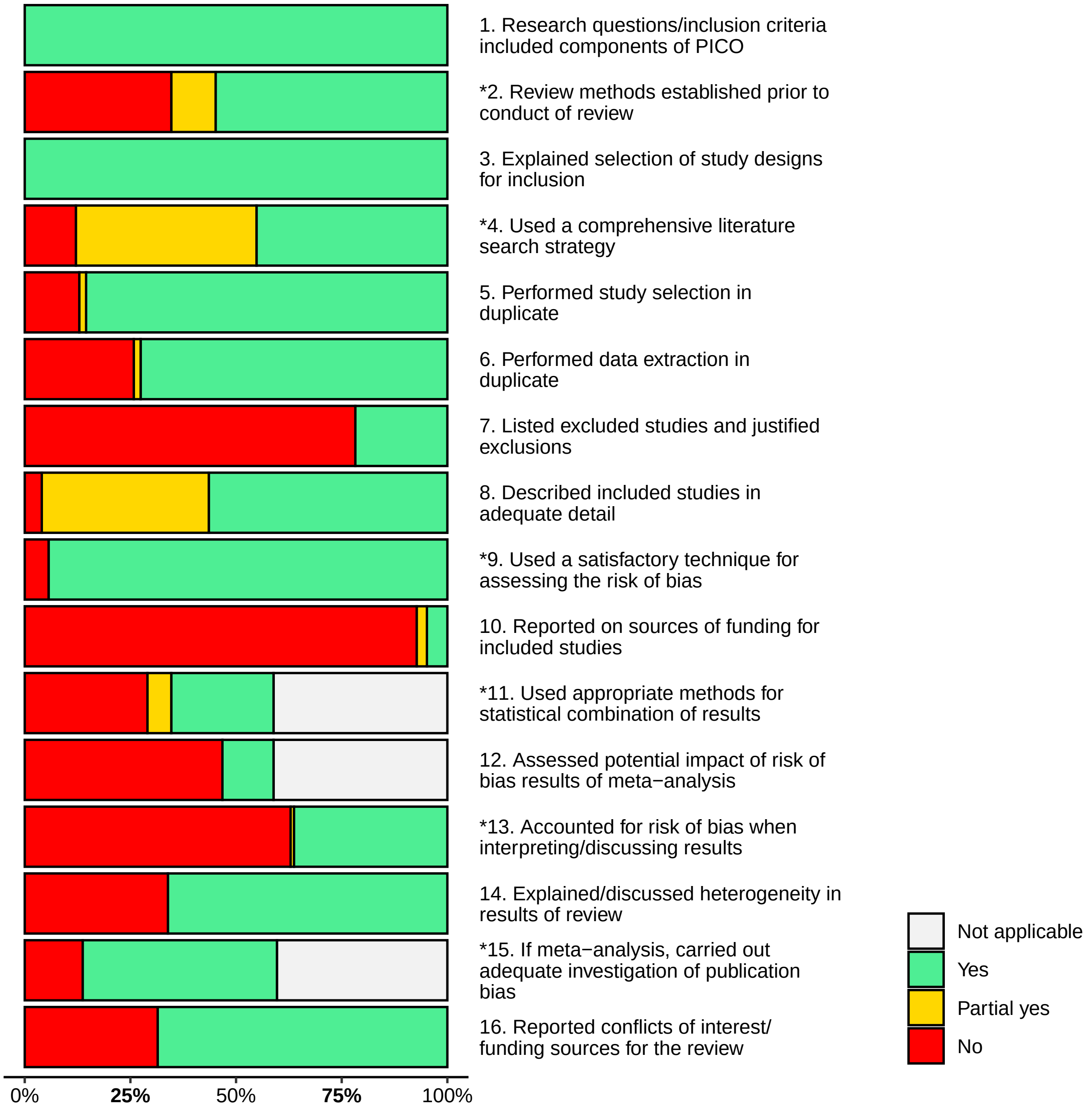

* Critical item.

Supplementary Figure 2: Distribution of risk-of-bias judgements in each bias domain for studies assessed using ROBINS-I within the included systematic reviews, stratified by the AMSTAR 2 confidence rating of the review

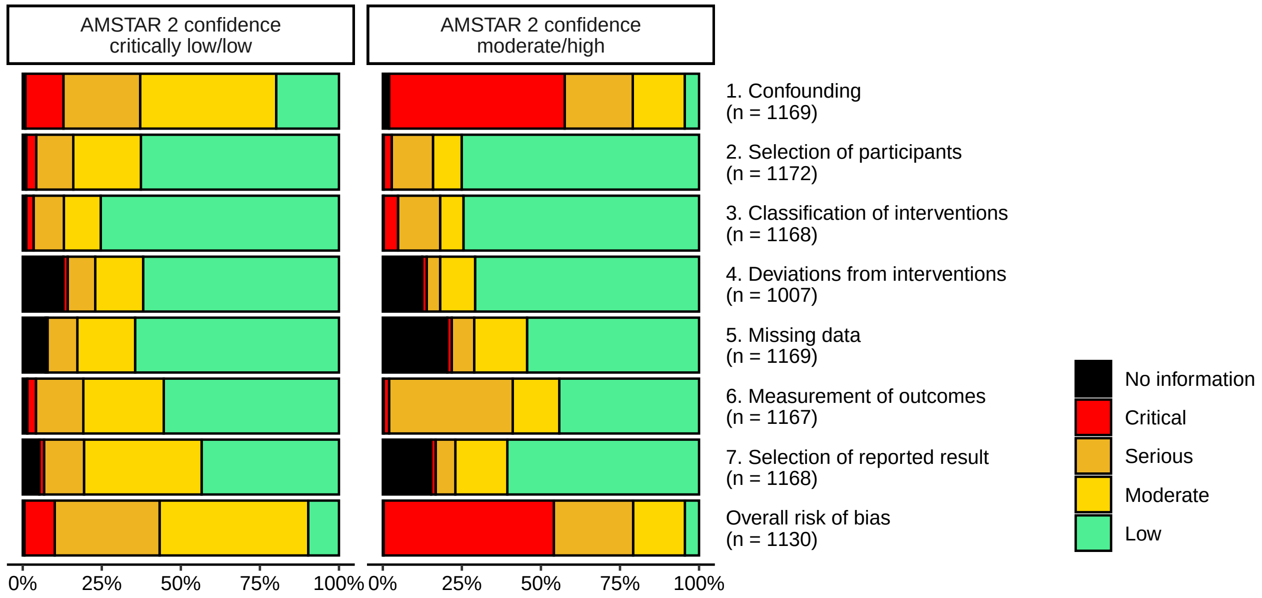

Supplement: Supplementary file 1 [file mmc1.pdf]
